# Immortalization of primary microglia: a new platform to study HIV regulation in the central nervous system

**DOI:** 10.1007/s13365-016-0499-3

**Published:** 2016-11-21

**Authors:** Yoelvis Garcia-Mesa, Taylor R. Jay, Mary Ann Checkley, Benjamin Luttge, Curtis Dobrowolski, Saba Valadkhan, Gary E. Landreth, Jonathan Karn, David Alvarez-Carbonell

**Affiliations:** 1 Department of Molecular Biology and Microbiology, Case Western Reserve University, Cleveland, OH 44106 USA; 2 Department of Neurosciences, Case Western Reserve University, Cleveland, OH 44106 USA; 3 Department of Biochemistry, Case Western Reserve University, Cleveland, OH 44106 USA

**Keywords:** Microglia, HIV regulation, HIV-associated neurocognitive disorders, NeuroAIDS

## Abstract

The major reservoirs for HIV in the CNS are in the microglia, perivascular macrophages, and to a lesser extent, astrocytes. To study the molecular events controlling HIV expression in the microglia, we developed a reliable and robust method to immortalize microglial cells from primary glia from fresh CNS tissues and commercially available frozen glial cells. Primary human cells, including cells obtained from adult brain tissue, were transformed with lentiviral vectors expressing SV40 T antigen or a combination of SVR40 T antigen and hTERT. The immortalized cells have microglia-like morphology and express key microglial surface markers including CD11b, TGFβR, and P2RY12. Importantly, these cells were confirmed to be of human origin by sequencing. The RNA expression profiles identified by RNA-seq are also characteristic of microglial cells. Furthermore, the cells demonstrate the expected migratory and phagocytic activity, and the capacity to mount an inflammatory response characteristic of primary microglia. The immortalization method has also been successfully applied to a wide range of microglia from other species (macaque, rat, and mouse). To investigate different aspects of HIV molecular regulation in CNS, the cells have been superinfected with HIV reporter viruses and latently infected clones have been selected that reactive HIV in response to inflammatory signals. The cell lines we have developed and rigorously characterized will provide an invaluable resource for the study of HIV infection in microglial cells as well as studies of microglial cell function.

**Electronic supplementary material:**

The online version of this article (doi:10.1007/s13365-016-0499-3) contains supplementary material, which is available to authorized users.

## Introduction

Microglial cells are the resident macrophages of the brain and spinal cord. Because the vulnerable neural tissue is largely shielded from leukocyte infiltrates and antibodies, microglial cells are the major and practically the only line of defense in the CNS, where they perform a variety of functions which are carried out by different cell types in the periphery. Microglia provide tissue surveillance within the brain, searching primarily for degenerating neurons, pathogens (Gehrmann et al. 1995), and apoptotic cells (Eyo and Dailey 2013). They are also involved in cytotoxicity to fight neuronal infection by viruses and bacteria, which can induce collateral damage to neurons (Gehrmann et al. 1995). Although microglia are poor antigen-presenting cells during their resting state, upon activation, they can upregulate MHC class I/II proteins and become efficient antigen presenters to T cells infiltrating the brain in certain inflammatory conditions (Aloisi 2001). There is also substantial evidence that microglial cells actively participate in synaptic stripping, tissue repair, and extracellular communication (Harry 2013). Due to their role in the CNS, microglial cells are extremely sensitive to small physiological and pathological changes, in part due to their specialized potassium channels (Dissing-Olesen et al. 2007; Gehrmann et al. 1995).

The activation of microglia required to combat pathogens in the CNS is a double-edged sword. Hyper-activation of microglial cells can directly cause the chronic inflammation observed during neurodegeneration, mainly due to the excessive secretion of cytokines and chemokines (Streit 2006). Many devastating neurodegenerative conditions, including Alzheimer’s disease (Mandrekar-Colucci and Landreth 2010), Parkinson’s disease (Sanchez-Guajardo et al. 2013), and HIV-associated dementia (HAD) (Watkins and Treisman 2015), are strongly correlated with exacerbated microglial activation.

It is generally agreed that HIV-1 replication in the CNS is initiated from invading perivascular macrophages, and then progresses to infection of microglial cells and, to a lesser extent, astrocytes within the brain parenchyma (Churchill et al. 2009; Conant et al. 1994; Eugenin and Berman 2007; Gorry et al. 2003; Hazleton et al. 2010; Watkins and Treisman 2015; Wiley et al. 1986). HIV-1 can infect microglial cells via CD4 receptors and a variety of chemokine co-receptors including CCR3, CCR5, and CXCR4, with CCR5 being the most important of the three (He et al. 1997). For example, IL-4 and IL-10 facilitate entry and replication of HIV-1 in microglia through the upregulation of CD4 and CCR5 expression, respectively. HIV-associated dementia (HAD) as well as less severe conditions known as HIV-associated neurocognitive disorders (HAND) strongly correlates with microglia activation, presumably due to combined deleterious effects of viral proteins and cytokines on neurons (Rock et al. 2004; Watkins and Treisman 2015).

Here, we describe a robust method for establishing immortalized microglial cell lines from a wide range of species, including humans. The immortalized cells have microglia-like morphology, express key microglial surface markers, demonstrate appropriate migratory and phagocytic activity, and have the capacity to mount an inflammatory response characteristic of primary microglia. Importantly, these cells can be used to generate a stable cell lines latently infected with HIV proviruses. In a related manuscript, we provide a detailed characterization of the responses of microglial cells to inflammatory activation signals (Alvarez-Carbonell et al. 2016). These extensively characterized cell lines will provide important tools to study microglial cell function and the mechanics and dynamics of HIV transcription in the CNS.

## Methods

### Isolation of primary microglial cells

Fresh CNS cortical tissue was obtained from adult patients undergoing brain surgery at University Hospitals of Cleveland (Human Tissue Procurement Facility, UH). Fresh mouse brain cortical tissue was obtained from newborn mice (CWRU). For effective manual dissociation of brain tissue, we used the Neural Tissue Dissociation Kit (P) (Miltenyi Biotec), following the manufacturer’s instructions. Dissociated cells (from approximately 1 g from human tissue or 400 mg from mouse tissue) were incubated with CD11b Microbeads (Miltenyi Biotec) for standard magnetic cell sorting. Isolated CD11b^+^ cells were then resuspended in DMEM:F12/10% FBS medium and cultured for 7 days prior to further treatment. Maturation of the primary cellular culture was monitored by phase-contrast microscopy.

### Infection of primary human microglia with HIV and primary macaque microglia with SIV

Primary human microglial cells, cultured in coversli ≤ chamber slides, were infected with replication competent R5 HIV (AD8gNef-GFP). After 4 days, the virus was removed and 10 μM Raltegravir was added for 72 h. The cells were then fixed (4% para-formaldehyde) and permeabilized (0.1% Triton X-100). Cells were then stained with DAPI and Alexa Fluor® 647-conjugated phalloidin (for actin detection; ThermoFisher Scientific), and imaged by Delta vision deconvolution microscopy to detect GFP expression (HIV), nuclei, and actin.

Similarly, primary macaque microglial cells, kindly provided by Dr. Janice Clements (JHU), were thawed and plated onto coverslip chamber slides. After a week in culture, cells were infected with replication competent SIV 17E-Fr particles. In this case, since this virus lacked the GFP-reporter, SIV expression was measured by immunostaining for the SIV p27 gag protein using a SIVmac p27 monoclonal antibody (55-2F12; NIH AIDS Reagents Program) followed by anti-mouse secondary-Alexa Fluor® 488-conjugated antibody (ThermoFisher Scientific).

### Immortalization of primary microglial cells

Primary microglial cells isolated from human, macaque or mouse brain, or cryopreserved human microglia (Sciencell, Cat. #1900) were infected with vesicular stomatitis virus G envelop simian virus 40 large T antigen viral particles (VSVG SV40), containing the pBABE-puro SV40 LT construct (Addgene, Plasmid #13970) by spinoculation. Transformed microglial cells were allowed to expand in the presence of 2 μg/mL of puromycin (selection antibiotic), and antibiotic-resistant colonies were selected.

A fraction of the SV40-immortalized cells, as well as CHME-5 microglial cells, (Janabi et al. 1995) were superinfected VSVG hTERT-neomycin viral particles containing the pBABE-neo-hTERT construct (Addgene, Plasmid #1774) in order to express human telomerase reverse transcriptase. Infection was also carried out by spinoculation, and antibiotic-resistant colonies were selected in the presence of 2 μg/mL puromycin and 600 μg/mL neomycin, or 600 μg/mL neomycin in the case of CHME-5-hTERT cells (hT-CHME-5).

### Species confirmation using CycT1

DNA from immortalized human microglial cells (hμglia) was isolated using the DNeasy Blood and Tissue Kit (Qiagen), and the CYCT1 gene amplified using the human CYCT1-specific primers Fwd 5′-TCC AGA ACT TCC AGT GTT GC-3′ and Rvs 5′-TGC TTC TGG GAA ATA AAT GC-3′, which yields a 500-Kb product. As a control, DNA from hT-CHME-5 cells was isolated and the CYCT1 gene amplified with the rat CYCT1-specific primers Fwd 5′-ACA GGG AAA CAG TCC ACC AG-3′ and Rvs 5′-TAT GAT TTA TCT GAT AGT-3′, which yield a 400-Kb product. Similarly, CYCT1 from macaque microglial cells was amplified for purified DNA and macaque CYCT1-specific primers Fwd 5′-ACA GGG AAA CAG TCC ACC AG-3′ and Rvs 5′-TAT GAT TTA TCT GAT AGT-3′. In each case, we used the Phusion Flash High-Fidelity (ThermoFisher F548L) polymerase, and the following PCR program: initial denaturing at 98 °C for 10 s, 30 cycles of denaturing at 98 °C for 1 s, annealing at 62 °C for 5 s, and extension at 72 °C for 15 s/Kb of product, and a final extension step at 72 °C for 1 min.

### Expression of microglial cell surface markers

Surface expression of microglia specific markers was detected by fluorescence microscopy. Cells were cultured on glass coverslips, fixed, permeabilized, and incubated for 1 h with biotin-anti-human CD11b (BioLegend 301304), or FITC-anti-human CD14 (BD 555397), or anti-P2RY12 (Abcam ab86195) or biotin-anti-human P2RY12 (Bioss bs-12072R) antibodies. Alexa Flour 647 mouse anti-GFAP (BD Pharmingen 560298) was used as negative control. Cells were then washed three times and incubated for 1 h with the secondary antibodies PE Streptavidin (BioLegend 405203) or Alexa Flour® 647 goat anti-rabbit (life technologies A21244) followed by exposure to DAPI-containing washing solution for nuclear staining, and fluorescence exposure for imaging.

Flow cytometry analysis (FACS) was performed using the LSR Fortessa instrument for cell sorting, the FACSDiva software (BD, NJ) for data collection, and the WinList 3D software for data analysis was used to measure surface expression of CD11b, CD14, CD68 (eBioscience 12-0689), CD16 (eBioscience 12-0167), CD32 (eBioscience 17-0329), CD64 (eBioscience 8012-0649), CD163 (eBioscience 12-1639), P2RY12, and TGFβR (Millipore ABF17). In addition, we also measured surface expression of CCR5 (BD 556889) and CD4 (BD 556615) in primary astrocytes as well as clones 1A1 and C20, and monitored the expression of these receptors across four passages (first, second, sixth, and tenth). For FACS analysis, we used 1 × 10^5^ cells resuspended in 1 mL of cold PBS in the presence of 0.5 μg of the antibody for 1 h at 25 °C. Appropriate secondary antibodies were used in the absence of fluorophore-conjugated primary antibody. Cell-antibody complexes were centrifuged, and the pellet resuspended in 300 μL of PBS before FACS analysis.

### Microglial cell migration and phagocytosis

Migration of hμglia cells was measured by monitoring cell movement on the culture plate surface during a period of 10 h in time-lapse experiments, using an automated, computer-controlled stage encoder, Leica DMI 6000B scope. Briefly, cells were plated at a density of 2.5 × 10^4^ cells per well in a 24-well plate, and pictures were taken every 30 min on pre-selected fields (8 fields total). Time-lapse movies were produced using MetaMorph® image analysis software (Molecular Devices, Downington, PA). The traveled distances of all the cells within the 8 fields were averaged, and the numbers plotted in a distance vs. time graph.

Similar time-lapse experiments were conducted in the presence of dead neuronal cells obtained after treatment of neurons with 0.05% trypsin, followed by 1–3 min vortex, in order to evaluate the phagocytic capacity of the hμglia cells. Cell death was verified by propidium iodide staining; at least 90% of cells were positive. Brightfield images were produced to count the number of dead neuronal cells present in the field as a function of time. The numbers were plotted in a number of dead neurons vs. time graph.

### Cytokine production

A representative line of hμglia cells (clone C20) was untreated or treated with TNF-α (10 pg/mL) for 16 h prior to collection of the supernatant and isolation of total RNA (see below). The supernatants were tested on Quansys Biosciences’s (Logan, UT) Q-Plex Array™ kit (human) for secretion of IL-1α, IL-1β, IL-2, IL-4, IL-5, IL-6, IL-8, IL-10, IL-12p70, IL-13, IL-15, IL-17, IL-23, IFNγ, TNF-α, TNF-β, Eotaxin, GROα, I-309, IP-10, MCP-1, MCP-2, RANTES, TARC, Ang-2, FGF, HGF, PDGF, TIMP-1, TIMP-2, VEGF, CD-163, Fractalkine, GM-CSF, and TGF-β. Absolute values of detected protein secretion were quantified in picograms/milliliter, from which the fold change expression was calculated.

### RNA-seq analysis

RNA sequencing (RNA-seq) was used as a tool to profile and confirm the microglia phenotype of clone C20, an immortalized human microglia cell line, in order to further validate our method to develop models of immortalized microglial cells. Total cellular RNA was used for preparation of RNA-seq libraries using Illumina TruSeq stranded Total RNA with Ribo Zero Gold kit, which includes removal of both cytoplasmic and mitochondrial ribosomal RNAs. Sequencing was performed on an Illumina HiSeq 2500 instrument at a depth of ∼35 million or more paired-end, 100-bp-long, strand-specific reads per sample. The resulting reads were quality controlled with fastqc (http://www.bioinformatics.babraham.ac.uk/projects/fastqc/), and low-quality reads were removed from the library using Trim Galore (http://www.bioinformatics.babraham.ac.uk/projects/trim_galore/). The reads that passed the quality filter were pseudoaligned to the Gencode V23 human transcriptome, and the reads that mapped to each transcript were quantitated using Kallisto (Bray et al. 2016) with 100 rounds of bootstrapping. Differential expression tests were performed with Sleuth (http://pachterlab.github.io/sleuth/) using the Wald test (SRA accession number SRP075430). Differentially expressed protein-coding transcripts showing twofold or more change in expression were used in pathway analysis using the GSEA pathway analysis tool (Subramanian et al. 2005) on the Hallmark (50 pathways represented) and Kegg gene sets subset (186 pathways represented) of the curated gene sets (C2) collections of the mSigDB v5 databases using 1000 permutations. Pathway terms containing less than 2 or more than 500 genes were eliminated from the pathway analysis. Pathways that show enrichment with a *p* value <0.05 and the stringent familywise error rate (FWER) of <0.25 were considered significant (Subramanian et al. 2005).

Single cell RNA-seq reads from the study of human brain cells by Darmanis et al. (Darmanis et al. 2015) was downloaded from NCBI SRA (accession no. GSE67835). Each file corresponding to an individual cell was checked for quality, aligned to the transcriptome and quantitated as described above. The alignment was performed both at the level of individual cells and after concatenation of the files to generate read volumes comparable to our bulk RNA-seq to improve the accuracy of analysis in direct comparison tests. RNA-seq reads from the analysis of mouse brain cells by Zhang et al. (Zhang et al. 2012) were downloaded from NCBI SRA (accession number GSE52564) and analyzed as described above. Heatmaps were generated by means of the Hierarchical Clustering Heatmap Python recipes (http://code.activestate.com/recipes/578175-hierarchical-clustering-heatmap-python/), using tpm (transcripts per million) values. Protein-coding transcripts that were expressed at 5 tpm or higher in at least one of the cell types under study were included in the analyses in Fig. 8. For the middle and bottom panels of Fig. 8a, genes with over twofold higher expression in human and mouse neurons versus astrocytes and microglia (middle panel) or genes enriched in astrocytes when compared to neurons and microglia (lower panel) were selected as neuron-enriched and astrocyte-enriched genes, respectively. Genes enriched in C20 cells versus human oligodendrocytes and astrocytes (middle panel) or versus human oligodendrocytes and neurons (bottom panel) were selected for comparison and their overlap with neuron- and astrocyte-specific genes were shown as Venn diagrams.

### Characterization of immortalized mouse microglial cells

Representative clonal populations of immortalized microglial cells derived from mouse brain (muμglia) were characterized by detection of CD11b and Iba1 protein expression as well as by detection of CD11b, Iba1, P2RY12 and TGFβR mRNA expression, and LPS-mediated inflammatory responses. For immunohistochemistry, cells were plated onto glass coverslips in a 24-well plate at a density of 100,000 cells/well. After 24 h, cells were switched to serum-free media. Following a 10-min fixation in 4% paraformaldehyde (PFA) with calcium and magnesium, cells were permeabilized in PBS pH 7.4 with 0.2% Triton-X and 3% normal donkey serum (NDS) for 1 h. Cells were blocked in PBS pH 7.4 with 3% donkey serum, 0.5% BSA and 0.2% Triton-X. Primary antibodies—rabbit anti-Iba1 (Wako) or rat anti-CD11b (ABD Serotec)—were added at a 1:500 dilution and cells incubated overnight at 4 °C. After washing, Alexa secondary antibodies were added at a 1:500 dilution for 1 h at room temperature. Samples were coverslipped using Prolong Gold and imaged on a Leica DM5000B scope.

The constitutive expression of CD11b (Mm00434455_m1), Iba1 (Mm00479862_g1), P2RY12 (Mm01289605_m1), and TGFβR (Mm03024091_m1) transcripts was measured by qPCR using the expression of each gene from one reference cell line as control. The mRNA expression of the pro-inflammatory markers IL-1β (Mm00434228_m1), IL-6 (Mm00446190_m1), and TNF-α (Mm00443258_m1) upon stimulation with 1 μg/mL of LPS was also detected by qPCR. For this, cells were plated in 6-well plates at a density of 500,000 cells/well and switched to serum-free media after 24 h. Cells were washed with PBS, lysis buffer (Ambion) was added to each well, and cells were removed using a cell scraper. Cells were passed through a 20G syringe ten times. Equal volume of 70% ethanol was added to each sample and the Ambion® RNA purification kit used to isolate RNA. Samples were treated with an on-column Purelink DNase kit (Life Technologies) according to kit instructions. Five hundred micrograms of RNA was converted into cDNA using a High Capacity RNA-to-cDNA kit (Applied Biosystems). Samples were pre-amplified for genes of interest using the Taqman Preamplification kit and gene expression assessed using Taqman Assays on a StepOne Plus. Gene expression levels were measured relative to GAPDH and normalized to the reference cell line to assess relative transcript levels across samples.

### Development of HIV latency models in microglia

HIV infection of hμglia was carried out as previously described for CHME-5 (Wires et al. 2012) to obtain clonal populations of hμglia/HIV cells. Briefly, infection by spinoculation was carried out with vesicular stomatitis virus G-(VSVG) pseudotyped HIV particles bearing a fragment of HIV-1_pNL4-3_, containing *Tat*, *Rev*, *Env*, *Vpu*, and *Nef* (some cell lines contain an older HIV construct carrying no *Nef* (Pearson et al. 2008) cloned into the pHR′ backbone together with the reporter gene 2dE green fluorescence protein (GFP), as previously shown (Dull et al. 1998; Pearson et al. 2008); (Fig. 11a). The viral particles were produced by the triple transfection of 293 T cells using lipofectamine, and the vector titer was determined as described previously (Kim et al. 2006b). GFP^+^ cells were isolated 48 h post-infection by fluorescence-activated cell sorting (FACS), further cultured, expanded, and allowed to enter into a latent state. Evaluation of HIV latency was performed by treatment with indicated doses of TNF-α, IL-1β, or LPS. To keep the levels of HIV basal expression low, cells were maintained in 1% FBS (in DMEM supplemented with 1X normocin).

## Results

### Infection of primary human and macaque microglia with HIV and SIV

**Fig. 1:**
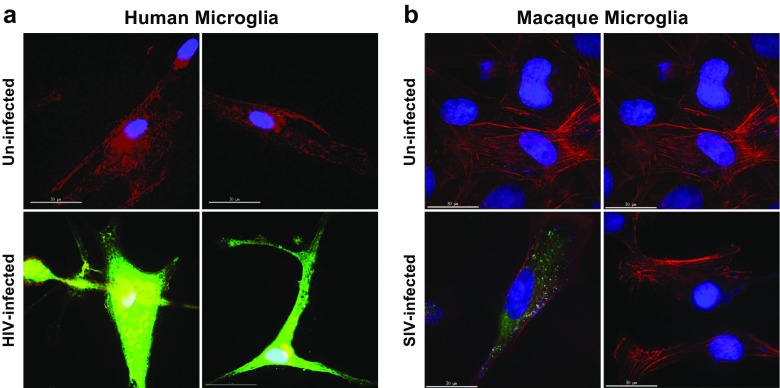
Infection of primary microglia cells with replication competent HIV viruses. **a** Primary human microglial cells were cultured onto coverslip chamber slides and incubated with replication competent R5 HIV (AD8gNef-GFP) for 4 days prior to viral removal and addition of 10 μM Raltegravir for 72 h. The deconvolution microscopy images are fixed and permeabilized cells stained with DAPI and Alexa Fluor® 647-conjugated phalloidin (for actin detection). GFP expression (HIV) is shown in *green*. **b** Primary macaque microglial cells plated onto coverslip chamber slides were incubated with replication competent SIV 17E-Fr virus. SIV expression was determined by immunoblotting for the SIV p27 gag protein using a SIVmac p27 monoclonal antibody followed by anti-mouse secondary-Alexa Fluor® 488-conjugated antibody

Although it is generally accepted that microglial cells are major sources of long-term HIV replication in the brain (Watkins and Treisman 2015), only a limited number ex vivo studies of the susceptibility of primary microglial cells to HIV infection have been performed (Jordan et al. 1991; Lee et al. 1993). It is particularly important to understand the susceptibility of microglial cells to HIV infection because peripheral macrophages are poorly infected by HIV due restrictions imposed by the SAMHD1 protein (Hrecka et al. 2011). To confirm that CD11b^+^ cells isolated from adult human brain are a direct target of viral infection ex vivo, primary cell cultures were infected using replication competent R5 HIV (AD8gNef-GFP). Our results demonstrated that HIV was expressed (GFP fluorescence) 5 days post-viral infection, as evidenced by high-resolution fluorescence microscopy (Fig. 1a) in approximately 75% of the cells in the culture.

In a separate experiment, infection of primary macaque microglial cells, kindly provided by the laboratory of Janice Clements (Baltimore, MD), by replication competent SIV 17E-Fr was also demonstrated (Fig. 1b). Similarly, expression of SIV was detected by immunoblotting for SIV p27 gag (green) in approximately 70% of the cells. In both experiments, cell nuclei were detected by Hoescht staining of DNA (blue), and cell shape by immunostaining for F-actin (red). The ramified morphology of the microglial cells was evident in both cultures (Fig. 1a, b).

### Isolation and immortalization of microglial cells from human and mouse brain

It is extremely difficult to perform molecular experiments in primary human microglial cells due to their rarity, the difficulty of obtaining surgical specimens, and their short lives in cultures. We therefore developed protocols to obtain immortalized cell clones that recapitulated many of the phenotypes of primary microglial cells.

**Fig. 2:**
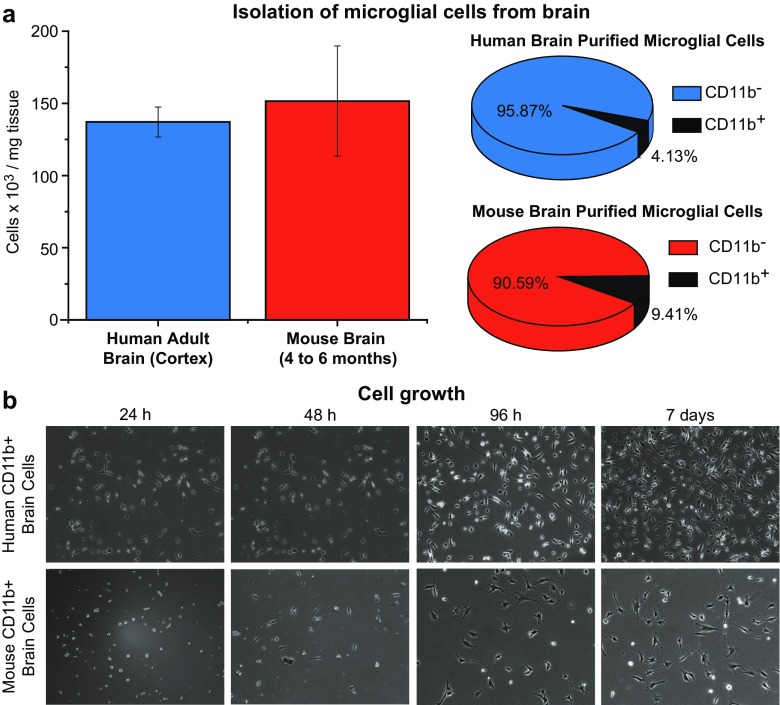
Isolation of CD11b^+^ cells from brain cortical tissue. **a** Fresh CNS cortical tissues from adult human patients undergoing brain surgery or fresh mouse brain cortical tissues were used as source of brain cells. The graph represents the average number of brain cells dissociated per milligram of brain tissue of either human (*blue*) or mouse (*red*) origin using the Neural Tissue Dissociation Kit (P). The *error bars* represent the variation of different isolations. **b** Dissociated cells were incubated with CD11b Microbeads (Miltenyi Biotec) and CD11b^+^ cells isolated by standard magnetic cell sorting. The pie graphs show the proportion of CD11b^+^ cells (*black*) in the total population of brain cells dissociated. The CD11b^+^ portion shown is an average of the three isolations. **c** CD11b^+^ cells were cultured in DMEM:F12/10% FBS medium for up to 7 days prior following treatment. The microphotographs show the visual maturation of the primary cellular cultures followed by phase-contrast microscopy

Human microglia populations were purified from cortical CNS tissue extracted from adult patients (Fig. 2). In three representative isolations, we used an average of 986 mg of tissue, yielding an average of 136 million cells, or 137 thousand cells per mg (Fig. 2a), of which approximately 5.57 million, or 4.10%, were CD11b^+^ cells (Fig. 2b). Mouse microglia were purified from 4- to 6 month-old mice. Four representative isolations, averaging 215 mg of brain tissue, yielded an average of 33 million of total cells, or 152 thousand cells per mg (Fig. 2a), from which an average of 3.11 million, or 9.42% were CD11b^+^ cells (Fig. 2b). The yield of CD11b^+^ cells is in close agreement to previously reported estimated fraction of microglia in the brain (Herculano-Houzel and Lent 2005).

The vast majority of the CD11b^+^ cells, of both human and mouse origin, displayed a ramified morphological phenotype (Fig. 2c), which was accentuated with time (1 vs. 2 vs. 4 vs. 7 days). A ramified morphological phenotype is characteristic of microglia (Kettenmann et al. 2011). Ramified microglia are present in abundance in the brain parenchyma and constitute approximately 10–20% of the total population of glial cells in the adult (Banati 2003; Vaughan and Peters 1974).

**Fig. 3:**
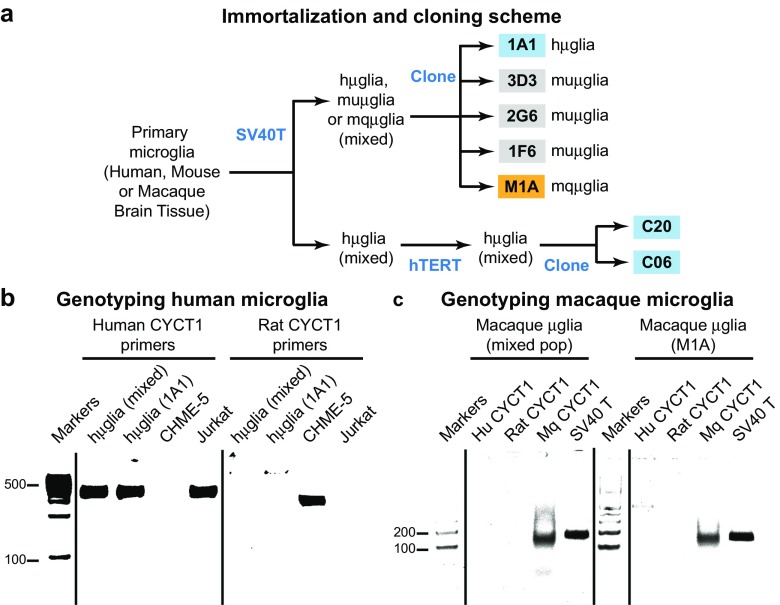
Preparation of clonal populations of microglial cells. **a** Schematic representation of a typical procedure to develop a microglia clonal cell population. Clonal populations are indicated in the *colored boxes*. Primary microglia from brain of different species are isolated and transformed with SV40 T-antigen. Clonal populations are either isolated from the mixed population or further transformed with hTERT antigen for clonal isolation. **b** Species origin of immortalized microglia is confirmed by amplification of the CYCT1 gene. The human CYCT1 gene is amplified with human-specific primers only from immortalized microglia derived from human sources (hμglia). DNA from Jurkat cells was used a positive control. Similarly, the rat CYCT1 gene is amplified with rat-specific primers only from cells derived from rat sources, as it is the case of CHME-5. **c** CYCT1 from macaque microglial cells was amplified for purified DNA and macaque CYCT1-specific primers. For these experiments, the Phusion Flash High-Fidelity polymerase was used, and the products loaded onto 1% agarose gels next to a ladder for estimation of band size

Primary microglia were transformed using either SV40 T antigen (SV40) or SV40/hTERT. A schematic representation of the procedure used to develop immortalized microglia is shown in Fig. 3a. In the course of this work, we developed a wide range of clonal cell populations from human (hμglia 1A1, C20, and C06), mouse (muμglia 3D3, 2G6, and 1F9) and macaque (mqμglia M1A) (Fig. 3a).

**Fig. 4:**
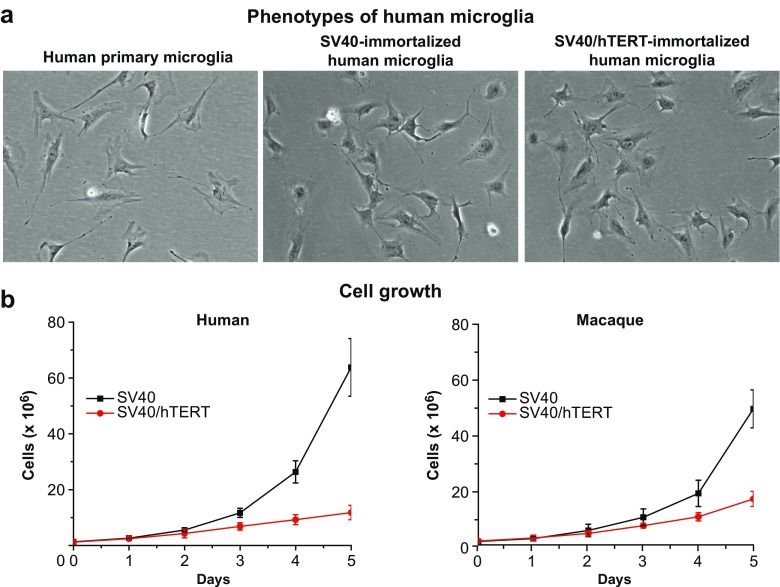
Immortalization of primary human microglial cells. **a** A phase-contrast microscopy photograph of human primary microglial cells is shown next to that of SV40- or SV40/hTERT-transformed cells. Immortalized microglia were allowed to expand in the presence of 2 μg/mL of puromycin (selection antibiotic for SV40 T antigen) or 2 μg/mL of puromycin plus 600 μg/mL of neomycin (selection antibiotic for hTERT). **b** The rate of expansion of SV40- vs. SV40/hTERT-immortalized human or macaque cells was monitored. The graphs represent the number of cells counted (*Y-axis*) from each culture once per day during 5 days (*X-axis*)

Two important observations were drawn from the immortalization experiments: First, immortalization-driven expansion of microglial cells did not compromise the typical phenotype observed in primary microglia cultures, as evidenced by the strong similaritires between primary and immmortalized cells (Fig. 4a). Second, the rate of expansion of immortalized cells was significantly diminished in the presence of hTERT, as evidenced by the slower doubling time captured in both human and macaque microglia immortalized with SV40 vs. SV40/hTERT (Fig. 4b). We have been unable to expand or maintain a culture of immortalized microglial cells using hTERT alone.

### CHME-5 cells are rat cells

Currently available transformed human microglial cell lines reported to be in use by other groups include HMC3 (Jadhav et al. 2014; Janabi et al. 1998; Janabi et al. 1995; Rawat and Spector 2016) and C13NJ (Martin et al. 2003). Both cell types are direct derivations of the original CHME-5 cells (Janabi et al. 1995), perhaps the most widely used microglial cell lines to study important aspects of HIV neuropathogenesis (Chugh et al. 2007; Jadhav et al. 2014; Janabi et al. 1998; Wires et al. 2012).

We have found to our surprise that CHME-5 are not human cells, as previously believed, but actually a transformed rat cell line. CHME-5 cells were originally created by transfecting human fetal microglia with the large T antigen of the simian virus 40 (SV40) (Janabi et al. 1995). We used CHME-5 cells to investigate HIV latency in microglia, and demonstrate cleavage of HIV proviruses by CRISPR-Cas9 (Hu et al. 2014). As we extended our studies to include CRISPR knockouts and ChIP assays, we began to obtain sequence information that suggested non-human sequences were present in the cells. Genotyping of the cells by microsatellite analysis (IDEXX BioResearch) also suggested that these cells might actually be of rat origin.

To identify the genotype of these cells unequivocally, we studied the expression of rat and human CYCT1 as well as SV40 T antigen using PCR assays. CYCT1, although highly conserved across mammals, bears important sequence variations, including deletions, which we have exploited to confirm the species of origin of the different cell clones.

As shown in Fig. 3b, distinct amplified CYCT1 fragments can be obtained from total DNA isolated cell lines of different species. Only PCR reactions including primers designed to target human CYCT1 gave a positive signal when DNA was purified from cells of human origin [hμglia (mixed pop), hμglia (clonal pop), and Jurkat, as positive control] (Fig. 3b). Similarly, primers designed to target CYCT1 of rat origin (CHME-5) (Fig. 3b), or CYCT1 of macaque origin (Macaque μglia (mixed pop) and Macaque μglia (clonal pop); Fig. 3c) produced unique fragments.

Originally, we obtained CHME-5 cells from the laboratory of Dr. Brandon Harvey (NIDA), with whom we published a study using these cells (Wires et al. 2012). After identifying these cells as rat cells, we obtained samples from laboratories that have reported to have used CHME-5 in the past, including those of Dr. Pierre Talbot (INRS, Canada), Dr. Marc Desforges (UQAM, Canada), Dr. Randall Davis (Oklahoma State University), and Dr. Jose Rodriguez (Universidad Central del Caribe, Puerto Rico), along with another sample from Dr. Harvey’s laboratory. All the CHME-5 cells received from these laboratories are of rat origin, suggesting that this was due to an early contamination of the original cell lines.

### Immortalized human microglia express characteristic surface markers

**Fig. 5:**
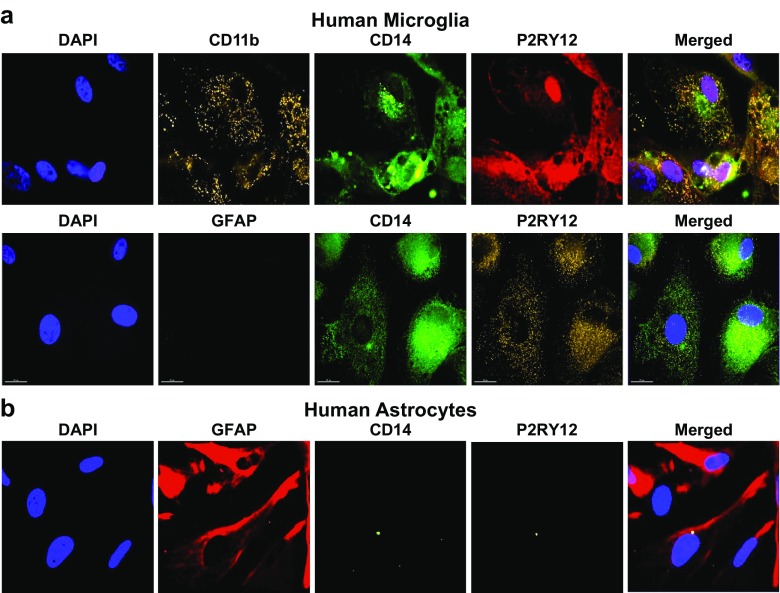
Expression of microglial markers in immortalized cells. **a** The expression of the microglial surface markers CD11b, CD14, and P2RY12 is shown by fluorescence exposure imaging (magnification ×60) in a culture of human primary microglial cells. **b** GFAP antibody, used as negative control, stained human astrocytes (magnification 40X). Microglial cells cultured on glass coverslips were fixed, permeabilized, and incubated with the respective antibodies for 2 h followed by washing with DAPI-containing washing solution for nuclear staining

Primary microglial cells typically express P2RY12 (Butovsky et al. 2014), which distinguishes them from peripheral macrophages. In agreement with this pattern of expression, we demonstrate that immortalized microglial cells derived from either fresh human brain tissue CD11b^+^ cells [hμglia (1A1) cells] or commercially available primary human microglia [hμglia (C06) cells] retained expression of CD11b (~46 and 67%, respectively), P2RY12 (~97 and 99%, respectively), and CD14 (~91 and 99%, respectively). In addition, these cells were negative for GFAP (Fig. 5a), which is expressed in astrocytes (Jacque et al. 1978) and ependymal (Roessmann et al. 1980) cells, but not in microglia (Fig. 5b).

In contrast to what we observed in primary microglia (Fig. 1), only a small fraction of cells (~1–5%) were infected by wild-type HIV in both 1A1 and C20 cells. To explain this loss of infectivity, we monitored the expression of CD4 and CCR5, the HIV recognition receptors in microglia (Kariko et al. 2005), in C20 cells during four sequential passages (first, second, sixth, and tenth) (Supplementary Fig. 1). CCR5 was detected on approximately 40% of the cells and remained stable across passages. By contrast, CD4 expression was low and decreasing with passage number (7.36%, first passage; 3.04%, tenth passage), consistent with the low fraction of infected cells we have observed.

**Fig. 6:**
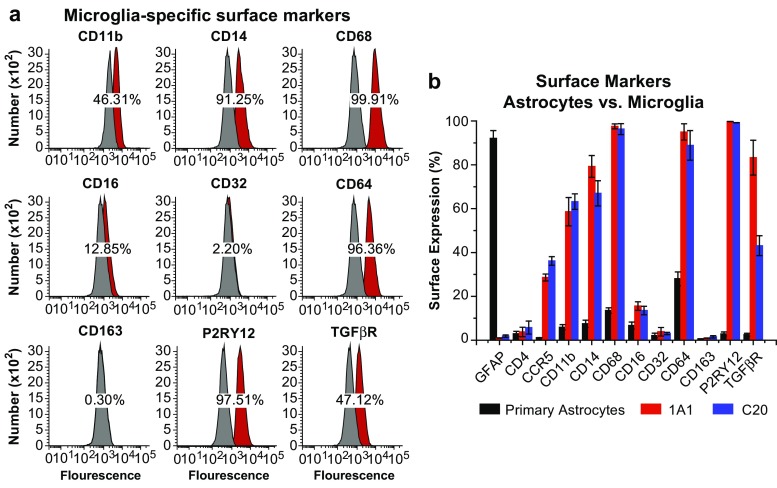
Surface expression of key markers of microglia. **a** Flow cytometry analysis was used to measure the surface expression of CD11b, CD14, CD68, CD16, CD32, CD64, CD163, P2RY12, and TGFβR on the representative immortalized cell line hμglia 1A1. In each experiment, 100,000 cells were resuspended in 1 mL of cold PBS in the presence of 0.5 μg of the antibody or isotype control for 20 min on ice. Appropriate secondary antibodies were used in the absence of fluorophore-conjugated primary antibody, and the cell-antibody complexes were centrifuged and resuspended in PBS. In each FACS profile, the *gray* distributions represent the proportion of cells bound to the isotype control, whereas the *red* distributions represent the proportions of cells bound by the target antibody. **b** Quantification of the abovementioned markers as well as GFAP, CD4, and CCR5 on the surface of primary human astrocytes and immortalized microglia, as indicated

To further characterize the phenotype of the 1A1 and C06 cells, we measured surface expression of informative surface markers by flow cytometry. As shown in Fig. 6a (1A1 cells), CD68, which together with CD11b and CD14 is a key marker of brain macrophage/microglia (Graeber et al. 1990; Slepko and Levi 1996), was present in this clone (~100%). The cells also expressed the IgG Fc receptors, key regulators of phagocytosis (Tuijnman et al. 1993): CD16 (~13%), CD32 (~2%), and CD64 (~96%). The presence of the Fc gamma receptors CD16, CD32, and CD64 has been reported on microglia from both healthy individuals and patients with neurodegenerative disorders (Peress et al. 1993; Walker and Lue 2015). CD163, which is specific for perivascular macrophages (Kim et al. 2006a), was not present in this clone. Finally, TGFβR which, together with P2RY12, is a specific marker of microglial cells (Butovsky et al. 2014) was also detected at significant level in 1A1 cells (~47%).

Quantification of these markers, together with CFAP, CD4, and CCR5, on microglial clones 1A1 and C20 and human primary astrocytes (Fig. 6b), confirmed that GFAP was expressed in astrocytes, but in microglia (~90 vs. 1–3%), whereas microglial markers were more significantly expressed in microglia, and less so in astrocytes (CD11b ~60–65 vs. ~7%; CD14 ~65–80 vs. ~8%; CD68 ~96–98 vs. ~14%; CD16 ~13–16 vs. ~7%; CD64 ~93–97 vs. ~30%; P2RY12 ~98 vs. ~ 6%; and TGFβ ~40–90 vs. ~5%). Expression of CD32 and CD163 was low (~0–3%) in both types of cells. As noted above, the levels of CD4 in microglia were practically as low as in astrocytes (~5–6 vs. ~3%), but those of CCR5 were markedly different (~30–40 vs. ~ 2%).

### Migration and phagocytosis of dead neuronal cells by microglial clones

**Fig. 7:**
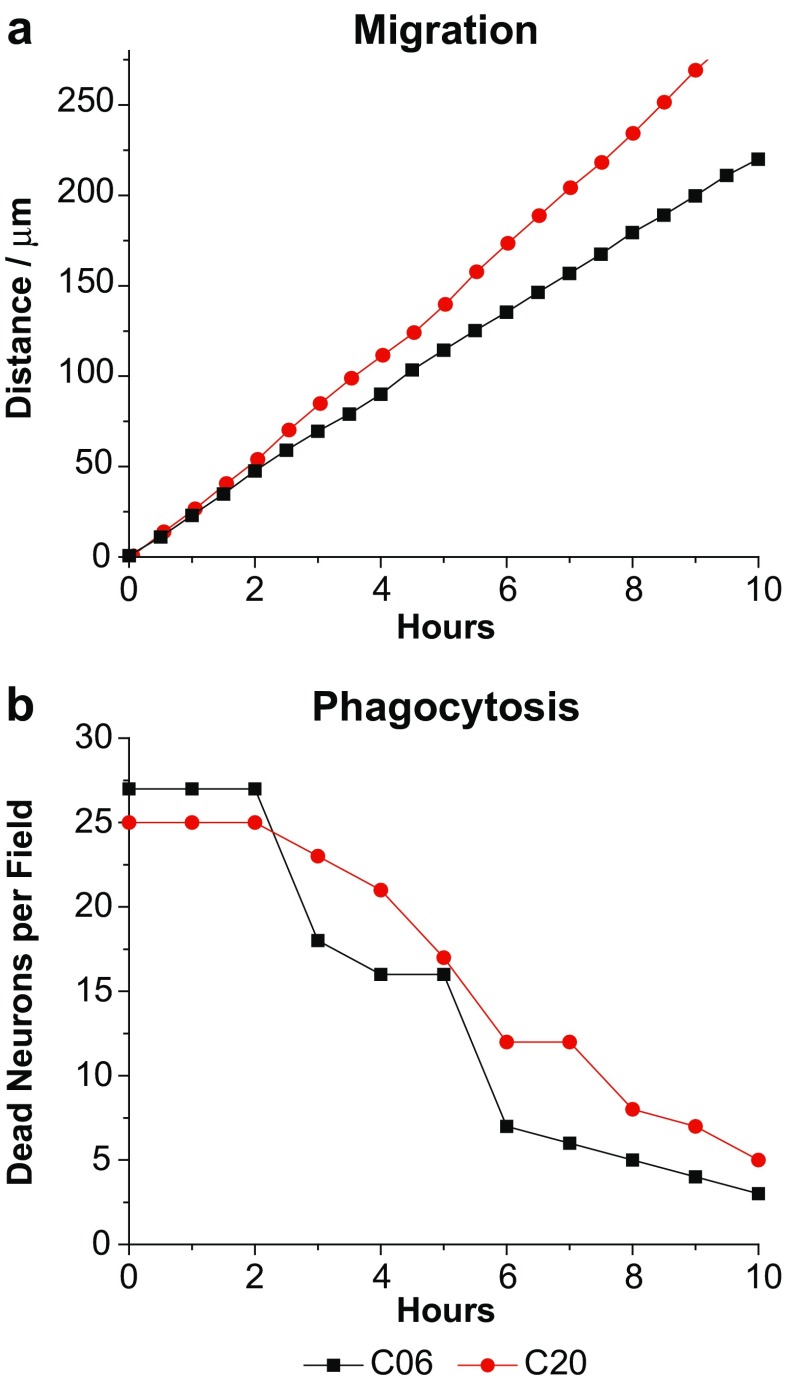
Functional features of immortalized microglial cells. **a** hμglia cell lines C06 (*black*) and C20 (*red*) were plated at a density of 1 × 10^5^ cells per well in a 24-well plate, and pictures were taken every 30 min on pre-selected fields (8 fields total). Time-lapse movies were produced using MetaMorph® image analysis software, and the distance traveled (in μm) by a single cell was determined by adding the distance the cell traveled along the surface. **b** Dead neuronal cells were added to the media of the indicated immortalized microglial cells, and the number of neurons remaining in the media was counted at the time points indicated. Dead neuronal cells were obtained by treatment of neurons with 0.05% trypsin, followed by 1–3-min vortex

A key microglial phenotype is their ability to migrate and phagocytose dead neurons. To evaluate how well-immortalized microglia retain these functional features, migration was measured in a 10 h cell culture by time-lapse, following individual cells and calculating the distance traveled by the cell during a 30-min period of time (Fig. 7a). The representative clones (C06 and C20) of immortalized human microglial cells were able to migrate around the culture plate at a speed of approximately between 20 (C06) to 27 (C20) μm/h, which appears to be similar to the migration rates of rabbit primary microglia (Rawat and Spector 2016; Wires et al. 2012).

We evaluated the ability of these clonal cell lines to phagocytose by adding dead neuronal cells to the culture media and counting the number of dead neurons that remained every 30 min after a period of 10 h (Fig. 7b). Both clones were capable of consuming dead neuronal cells. Interestingly, in both cases, no reduction in the number of dead neurons was observed during the first 2 hours, and this number precipitously fell during the next 8 h to approximately zero, suggesting a delay in developing the cellular phagocytic machinery.

### The gene expression profile of immortalized human microglia closely resembles that of primary microglia

To further confirm the microglial phenotype of the immortalized cells, clone C20 was untreated or treated with TNF-α for 16 h. The purified RNA from these cells was subjected to RNA sequencing, while the supernatant collected was used to quantify cytokine/chemokine secretion.

In order to determine whether the C20 cells (untreated or TNF-α-stimulated) retain the gene expression profile characteristic of primary microglia, the RNA-seq results obtained in our experiment were compared to the RNA-seq-derived gene expression patterns observed in the other major cell types, namely astrocytes and neurons, as well as microglia obtained from adult human temporal cortex, as described in Darmanis et al. (2015). An additional RNA-seq dataset that included astrocytes, neurons, and microglia from the mouse cerebral cortex (Conant et al. 1994) was also included in the analysis. To eliminate the biological and technical variability stemming from the species- and donor-specific differences and the use of single cell versus bulk RNA-seq, we defined the gene expression pattern specific to the microglia within each dataset as genes that showed at least twofold higher expression in microglia compared to neurons and astrocytes with a *p* value of less than 0.01. As we were not able to compare the gene expression pattern of the immortalized C20 cells with astrocytes and neurons derived from the same donor, we compared their gene expression pattern against pooled single-cell RNA-seq reads obtained from human astrocytes and neurons (Darmanis et al. 2015) (see “Methods” section).

**Fig. 8:**
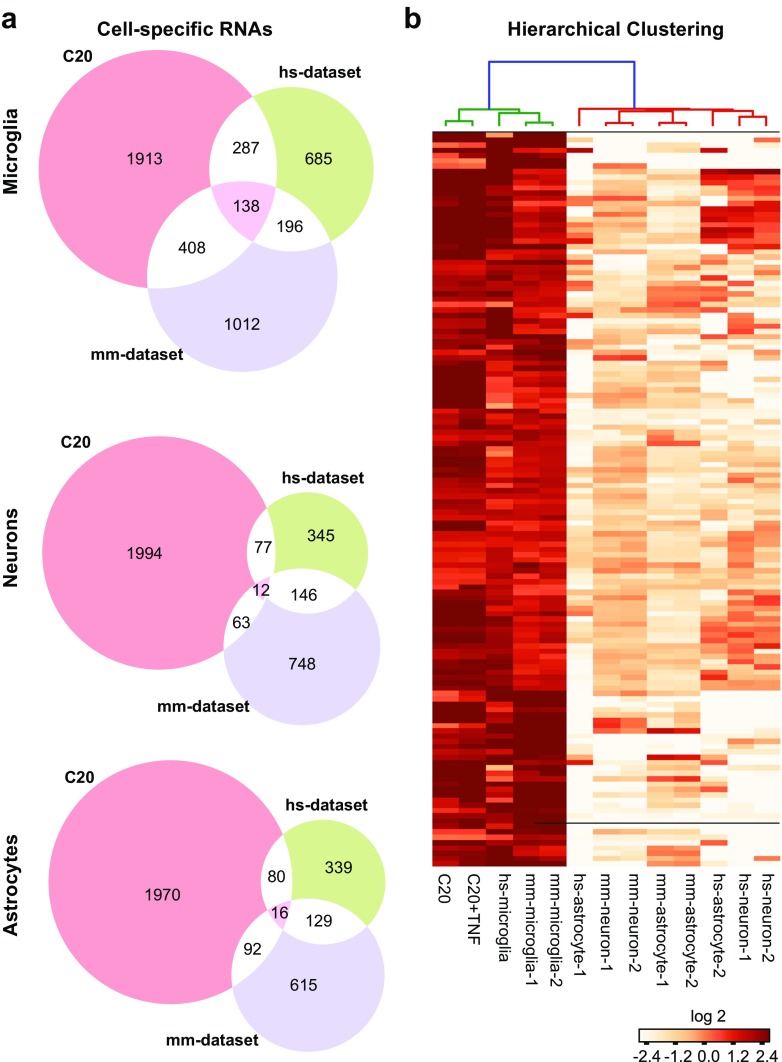
Immortalized human microglial gene expression profile relative to that of other brain cells. RNA-seq was used to confirm the microglial phenotype of the representative clone C20. The RNA-seq reads obtained were aligned to annotated reference genomes and counted to calculate abundance. **a** The relative number of identical genes (138) expressed in C20 as well as in primary microglia of a human set (*hs-dataset*) and of a mouse set (*mm-dataset*) was compared to the number of overlapping genes in neurons (12) or astrocytes (16) of the same sets. **b** Hierarchical clustering of genes expressed in C20 cells untreated or pre-treated with TNF-α with respect to genes expressed in indicated cells from the human or the mouse sets

As shown in the Venn diagrams of Fig. 8a, microglia-specific genes from C20 cells showed a high proportion of overlap with the genes identified in previous analyses of human and mouse microglia (Darmanis et al. 2015). Over 100 enriched genes were shared among the three microglial cells, including the TNF-treated C20 cells, which showed a similar enrichment of this set of “microglia-specific” genes (Fig. 8b). In contrast, C20 cells showed minimal overlaps with human or mouse neurons (Fig. 8a, middle panel) or astrocytes (Fig. 8a, lower panel, see “Methods” section).

### RNA-seq analysis of microglial inflammatory responses

For the RNA-seq analysis, the 1444 genes found at least twofold differentially expressed in C20 cells before and after treatment with TNF-α were subjected to pathway analysis using the Broad Institute software Gene Set Enrichment Analysis (GSEA) and the mSigDB Hallmark collection (see “Methods”).

As shown in Table 1, 6 out of the 50 gene sets were significantly (*p* ≤ 0.001) upregulated by TNF-α including the following: (i) the TNF-α signaling pathway via NF-κB (Kaltschmidt et al. 2005), our positive control; (ii) IFN-α (Khorooshi et al. 2015) response; (iii) IFN-γ (Tsuda et al. 2009) response; (iv) inflammatory response (Fonken et al. 2015); (v) IL6/JAK/STAT3 (Dominguez et al. 2008); and (vi) IL2/STAT5 signaling (Sheng et al. 2014).

**Table 1:** Sets of upregulated genes in C20 cells treated with TNF-α. List of top six pro-inflammatory pathways with nominal *p* ≤ 0.001. The number of genes of each pathway is also listed, together with a reference as an example of the pathway’s association with microglia activation in a neurodegenerative context

Gene sets significantly upregulated by TNF-α in clone C20 (GSEA analysis)	Number of genes	Example of gene set association with microglia and neurodegeneration/neuro-inflammation
TNFA_SIGNALING_VIA_NFKB	76	Kaltschmidt et al. 2005
INTERFERON_GAMMA_RESPONSE	99	Tsuda et al. 2009
INTERFERON_ALPHA_RESPONSE	66	Khorooshi et al. 2015
INFLAMMATORY_RESPONSE	49	Fonken et al. 2015
IL6_JAK_STAT3_SIGNALING	25	Dominguez et al. 2008
IL2_STAT5_SIGNALING	37	Sheng et al. 2014

These gene sets, or pathways, and their major components are characteristic of the microglial response to neuroinflammatory stimuli (Kaminska et al. 2015). For example, the Toll-like receptors, TLR2 and TLR3, ranked at 131 and 481 among genes upregulated in response to TNF-α, respectively, are part of the inflammatory response gene set (Table 1). The list of genes found in each of these 6 pathways is provided in Table 2.

**Table 2:** KEGG signaling pathways found up-regulated in C20 cells treated with TNF-α. List of top 6 pro-inflammatory pathways with *p* ≤ 0.001 for the first five pathways and *p* ≤ 0.003 for the last pathway. The number of genes of each pathway is also listed, together with a reference as an example of the pathway’s association with microglia activation in a neurodegenerative context

Pathways significantly upregulated by TNF-α in clone C20 (KEGG analysis)	Number of genes	Example of pathway association with microglia and neurodegeneration/neuro-inflammation
TNFA_SIGNALING_PATHWAY	37	Kaltschmidt et al. 2005
NF-KAPPA_B_SIGNALING PATHWAY	35	Kaltschmidt et al. 2005
CYTOKINE_CYTOKINE_RECEPTOR_INTERACTION	32	Kothur et al. 2016
CHEMOKINE_SIGNALING_PATHWAY	19	Kothur et al. 2016
NOD_LIKE_RECEPTOR_SIGNALING_PATHWAY	13	Kigerl et al. 2014
TOLL_LIKE_RECEPTOR_SIGNALING_PATHWAY	18	Kigerl et al. 2014

In addition to the Hallmark collection, we also use the Kyoto Encyclopedia of Genes and Genomes (KEGG) database resource to confirm that pro-inflammatory pathways characteristic of microglial cells were upregulated in TNF-α-stimulated C20 cells. The top 6 pathways that were significantly (*p* ≤ 0.003) upregulated are listed in Table 2. As expected, the top 2 pathways were our positive control pathways, TNF-α and NF-κB signaling pathways (Kaltschmidt et al. 2005), followed by the cytokine-cytokine receptor interaction and the chemokine pathways (Kothur et al. 2016), then the NOD-like receptor and the TLR signaling pathways (Kigerl et al. 2014). Activation of each one of these pathways has been strongly implicated in neurodegenerative disorders (Kaminska et al. 2015) (Table 2).

**Fig. 9:**
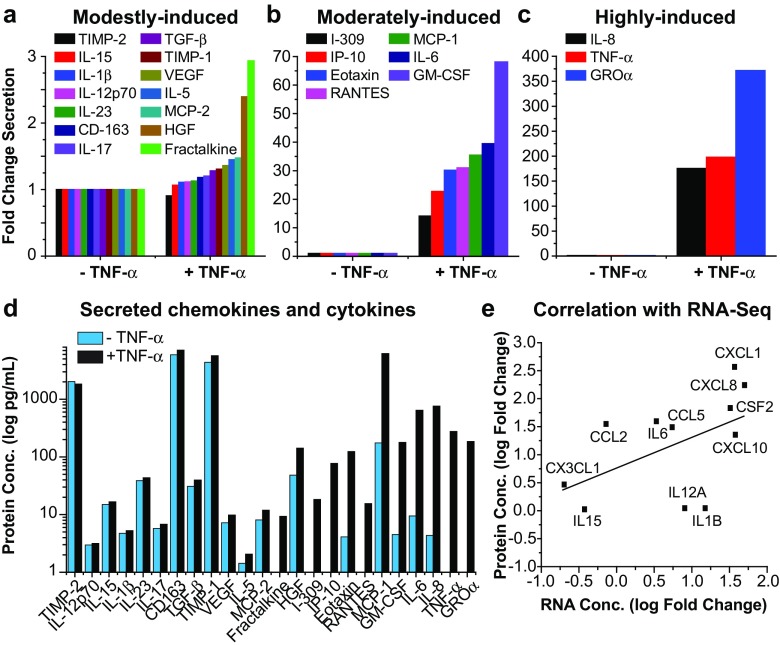
A representative line of hμglia, clone C20, produces cytokines in response to inflammatory stimuli. Clone C20 was treated with TNF-α (10 pg/mL) for 16 h, and the supernatant collected and tested on Quansys Biosciences’s (Logan, UT) Q-Plex Array™ kit (human) for secretion of various cytokines and chemokines. Detected protein secretion was quantified based on fold change expression, and its shown in these three graphs representing **a** the modestly induced proteins (1–3-fold change), **b** the moderately induced proteins (10–70-fold change), and **c** the highly induced proteins (150–400-fold change). **d** Concentration (in pg/mL) of secreted chemokines or cytokines in a logarithmic scale. **e** Correlation of RNA expression and protein secretion

We were able to experimentally verify the RNA-seq results by measuring cytokine release in response to TNF-stimulation. Of the 35 cytokines/chemokines that were measured, 24 were differentially secreted between untreated and TNF-α-treated cells (Fig. 9). The secretion of only one of these molecules, TIMP-2, was slightly downregulated, whereas the secretion of 13 others was upregulated between one- and threefold. These molecules, which included IL-15, IL-1β, IL-12p70, IL-23, IL-17, CD163, TGF-β, TIMP-1, VEGF, IL-5, MCP-2, HGF, and Fractalkine, were classified as modestly-induced (Fig. 9a). Moderately induced proteins, whose secretion was increased between 10- and 70-fold in the presence of TNF-α included I-309, IP-10, Eotaxin, RANTES, MCP-1, IL-6, and GM-CSF (Fig. 9b). Highly secreted proteins included IL-8, TNF-α, and GROα (Fig. 9c). The absolute values of detected secreted proteins are depicted in Fig. 9d.

We also compared the expression of TNF-α-induced genes (Fig. 8; Tables 1 and 2) with the levels of induced proteins (Fig. 9d). We found 11 pro-inflammatory molecules for which there was a high correlation between their induced RNA expression and protein secretion (Fig. 9e).

### Immortalized murine microglial cells resemble primary microglia

**Fig. 10:**
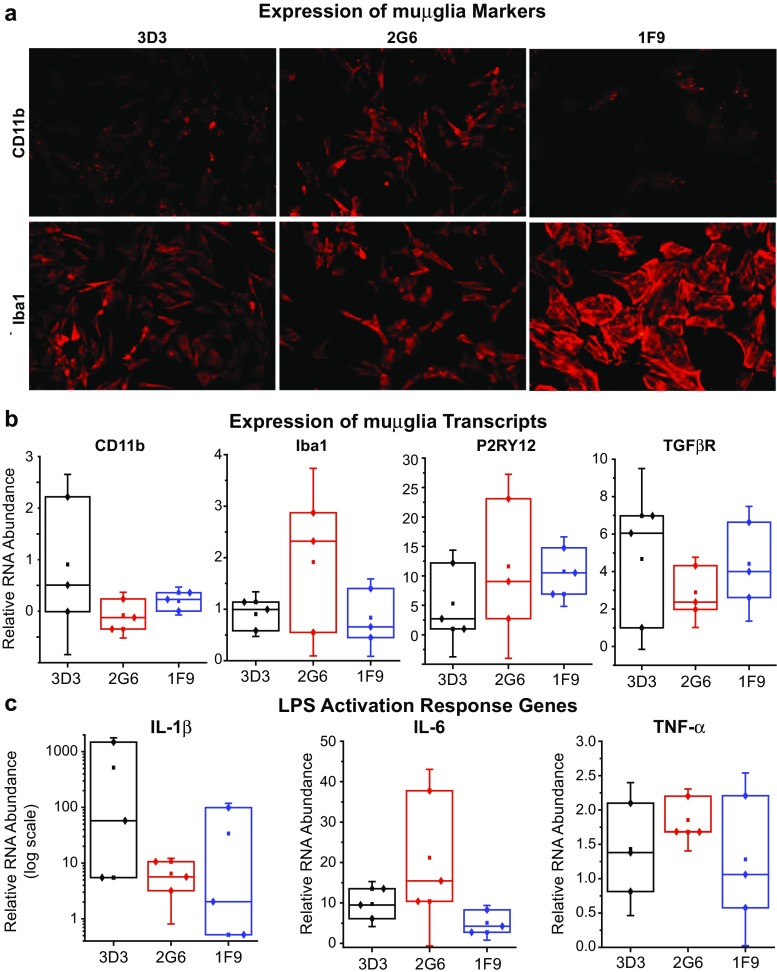
Immortalized mouse microglia displays key features of primary microglial cells. **a** Representative clonal populations of muμglia cells, 3D3, 2G6, and 1F9, express CD11b and Iba1 as shown by the immunocytochemistry microphotographs. 100,000 cells/well were fixed, permeabilized, and blocked, before incubation with the primary antibodies against CD11b or Iba1 at a 1:500 dilution. Alexa secondary antibodies were added at a 1:500 dilution before mounting and imaging. **b** Constitutive expression of CD11b and Iba1 RNA was detected in immortalized microglia by quantitative PCR. 500,000 cells/well were lysed and subjected to RNA purification followed by cDNA synthesis prior to amplification. The RNA sample from clone 3D3 was used as a reference. **c** Constitutive expression of P2RY12 and TGFβR was measured by quantitative PCR

An important advantage of our method for immortalizing microglial cells is that it is adaptable to cells from a wide variety of species. Clones from murine cells (muμglia) were assessed for protein expression of the myeloid cell markers CD11b and Iba1 by immunocytochemistry (Fig. 10a). CD11b and Iba1 transcripts were also detected in immortalized microglia by quantitative PCR (Fig. 10b). As expected, these immortalized cells also expressed transcript levels of the microglia-specific markers P2RY12 and TGFβR (Butovsky et al. 2014), though expression of these genes was more variable across three consecutive passage numbers (Fig. 10b).

Because microglia normally respond to LPS treatment by producing pro-inflammatory cytokines, we measured the expression of IL-1β, IL-6, and TNF-α following LPS treatment to assess whether microglial clones retained this functional response. In general, the clones responded to 24 h of LPS treatment by upregulating gene expression of these cytokines, though this was also variable across three consecutive passage numbers (Fig. 10c). Taken together, these results demonstrate that the immortalized microglial clones recapitulate some aspects of primary cultured microglial cells.

### Microglial cells latently infected by HIV are induced to stimulate viral transcription by inflammatory agents

**Fig. 11:**
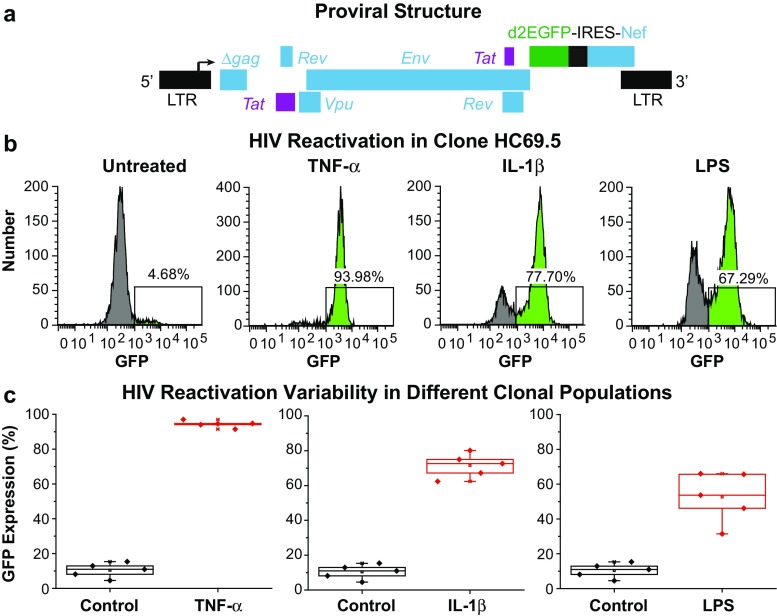
HIV emergence from latency in human models of infected microglia. **a** Genomic organization of the HIV lentiviral vector. A fragment of HIV-1_pNL4–3_, containing *Tat*, *Rev*, *Env*, *Vpu*, and *Nef* with the reporting gene d2EGFP, is cloned into the pHR’ backbone. The resulting plasmid was used to produce the VSVG HIV particles, as described previously (Kim et al. 2006b). **b** Flow cytometry analysis 16 h post-treatment using the representative clone HC69.5. GFP^+^ cell populations are indicated in *bright green*, and the percentage of GFP-expressing cells is indicated in *percentage*. **c** Reactivation of HIV in five different clonal populations: HC51, HC56, HC61, HC62, and HC69.5. TNF-α at 50 pg/mL, IL-1β at 100 pg/mL, and LPS at 1 ng/mL

Finally, we have developed various clonal models for hμglia latently infected by HIV. The hμglia cells were infected with vesicular stomatitis virus G-(VSVG) pseudotyped lentiviral vectors expressing Tat, Rev, Env, and Vpu, and carrying a short-lived green fluorescence protein (d2EGFP) (Fig. 11a) that allows monitoring of viral transcription by fluorescence-activated flow cytometry (FACS) and/or fluorescence microscopy (Wires et al. 2012). Saturating concentration of TNF-α (~94%), IL-1b (~78%), or LPS (~67%) stimulated HIV transcription, as shown in Fig. 11b, in the representative clone (HC69.5) derived from a mixed population of HIV-infected hμglia (Fig. 3). This and other similar clonal lines (HC51, HC56, HC61, and HC62), showing similar, but somewhat variable response to inflammatory stimuli (Fig. 11c), have proven valuable to study HIV latency regulation in microglia.

## Discussion

### HIV infection of microglial cells

It has been known since 1980s that the human brain harbors HIV-1 primarily in multinucleated giant cells and microglia (Kure et al. 1991; Navia et al. 1986; Sharer et al. 1986). Microglia are the major target cell type for HIV-1 infection in the CNS (Price et al. 1988; Takahashi et al. 1996; Watkins et al. 1990), with CCR3 and CCR5 providing the port of entry for macrophage-tropic viruses. Microglial cells express both CCR3 and CCR5, and He et al. (He et al. 1997) showed that the CCR3 ligand, eotaxin, and an anti-CCR3 antibody inhibited HIV-1 infection of microglia, as did MIP-1β, which is a CCR5 ligand. A similar mechanism has been described for infection of macaque microglial cells by SIV, specifically because SIV encephalitis is associated with the active replication of macrophage-tropic, neurovirulent viruses whose RNA predominated in the brain of macaques with encephalitis, particularly in their microglial cells (Babas et al. 2003). Early ex vivo studies demonstrated productive infection of primary microglia (Jordan et al. 1991; Lee et al. 1993). We have confirmed here that primary microglial cells isolated from either human or macaque brain were readily infected by replication competent HIV or SIV, respectively, and were capable of supporting viral replication (Fig. 1). Unfortunately, the same level of infection was not maintained in the immortalized cells. We believed that this is the result of a significant decrease of CD4 expression, and not of CCR5, after transformation. There are no blocks to reverse transcription, integration or expression in these cells since VSV-pseudotyped viruses readily infect them. Work is in progress to derive microglial lines that retain high levels of CD4 expression and support HIV replication.

### Immortalization of microglial cells

The extremely limited availability of primary microglial cells and their short lives in culture has precluded us from performing key experiments addressing fundamental questions about HIV expression regulation in brain. Compared to what we already know about the regulatory mechanisms of HIV expression in T cells, the knowledge accumulated in the realm of HIV infection in CNS, particularly in its regulation in microglia at the molecular level, is very scarce. Therefore, we believe that modeling ex vivo HIV infection and expression regulation in microglial cells will accelerate our understanding of the molecular basis of HAD and HAND.

The methods described here have enabled us to develop reliable cellular models of microglia by immortalization of primary glial cells from various species origins. In our method, we have used two immortalization agents: SV40 T antigen and hTERT. The SV40 T antigen, which is the most reliable agent for the immortalization of many different cell types in culture, works primarily by disrupting the normal function of retinoblastoma (pRB) and p53 tumor suppressor proteins in the cell, as well as by binding to transcriptional activators such as p300 and CBP (Ali and DeCaprio 2001). Therefore, it is reasonable to assume that SV40-mediated immortalization would induce certain tumorigenic features in the transformed cells. We therefore decided to use hTERT, alone or in combination with SV40 T antigen, to immortalize primary microglia. hTERT activity results in the maintenance of sufficient telomere lengths to avoid replicative senescence, and therefore seems to be a cleaner method for cell transformation. Indeed, strong evidence indicates that the hTERT enzyme can induce immortalization of human primary cells without causing cancer-associated changes or altering phenotypic properties (Jiang et al. 1999; Lee et al. 2004; Morales et al. 1999; Ouellette et al. 2000). Unfortunately, we have not been successful in maintaining a transformed microglial cell line that carries hTERT alone since the cells eventually stop growing and die. The best scenario we have devised is to use a combination of SV40 and hTERT, which, as shown in Fig. 4, delays the growth of the cells and leads to a less activated phenotype. We have not observed significant differences in surface marker expression between cells carrying SV40 alone, (hμglia 1A1 cells), and cells carrying both SV40 and hTERT (hμglia C06 cells) (Fig. 6).

We believe that the hμglia 1A1 cells are the first immortalized human microglial cell line of cortical origin. Nagai et al. (Nagai et al. 2005; Nagai et al. 2001) successfully immortalized human microglia from embryonic telencephalon tissue transformed by a retroviral vector encoding *myc* oncogene. This cell line has been used recently to identify TMEM119 as a reliable microglial marker that discriminates resident microglia from blood-derived macrophages in the human brain (Satoh et al. 2015).

The use of hTERT as part of the immortalization protocol makes our lines distinctive from the previously available SV-40-transformed human microglial cell lines, HMC3 (Jadhav et al. 2014) and C13NJ (Martin et al. 2003), which have not been as extensively characterized, and maybe be of rat origin, as stated above, because they derived from CHME-5 (Fig. 3). More recent attempts to model primary microglia without transformation agents have been reported. For example, a monocyte-derived microglia cell model of HIV infection was able to recapitulate infection of primary human microglia (Rawat and Spector 2016). A similar method was used to develop a model of human microglia (Etemad et al. 2012). In general, characterization of these monocyte-derived microglial cells extensively overlapped with our cell lines.

### Phenotypic characterization of microglial cell clones

Initial phenotypic characterization of our stable immortalized cell lines included detection of characteristic markers by immunofluorescence and flow cytometry (Fig. 6). CD11b has long been a marker used to identify microglia, although it is accepted that other CNS macrophages also express CD11b (Becher and Antel 1996; Butovsky et al. 2014; Ford et al. 1995). CD14, LPS co-receptor, is expected to be constitutively expressed in microglia (Etemad et al. 2012), and its expression has been found to increase in response to inflammation (Beschorner et al. 2002; Zhou et al. 2013). P2RY12 and TGFβR (Fig. 6) have been shown to be markers specific for microglia (Butovsky et al. 2014). Another important marker for microglia is CD68, shown to be present in our immortalized lines (Fig. 6), although it can also be found in macrophages (Graeber et al. 1990; Slepko and Levi 1996).

Expression of CD163 is more associated with perivascular macrophages than with microglia, although some reports suggest that this receptor is expressed in activated microglia (Kowal et al. 2011; Roberts et al. 2004; Zhang et al. 2012). The anti-CD163 antibody we used in our assay did not detect the presence of CD163 on the surface of either 1A1 or C06 cells (Fig. 6). However, we were able to detect very high levels of CD163 secreted by C20 cells, even in the absence of stimulation by TNF-α (Fig. 8) suggesting that the proportion of CD163 bound to the membrane is negligible compared to the secreted form of the receptor.

Iba1, which has been also used as a marker of activated macrophages/microglia (Schluesener et al. 1998; Schluesener et al. 1999), and very recently as a read-out for activation of microglia exposed to HIV Tat (Paris et al. 2015), was found present in the immortalized mouse microglia (Fig. 10a, b).

### Phagocytosis of dead neurons by microglial cells

Microglia exhibit pinocytotic activity and localized motility (Booth and Thomas 1991; Fetler and Amigorena 2005; Thomas 1992) consistent with the role of microglia as the brain immune scavengers (Fetler and Amigorena 2005). In CNS, microglia are highly motile, secrete inflammatory cytokines, migrate to the lesion area, and phagocytose cell debris or damaged neurons (Fu et al. 2014). We were able to verify the motility, or migratory capacity, of the immortalized cell lines C06 and C20 (Fig. 7). In order to determine whether the immortalized microglial cells retained their phagocytic phenotype, dead neurons were fed to microglia in culture. Dead neurons were sufficient to induce phagocytosis in microglial cells since neuronal death was induced in the absence of any agent that could have caused microglial activation, demonstrating that immortalized microglia retained their ability to detect dead or damaged neurons.

The phagocytic activity observed in both C06 and C20 cells is expected since we show that immortalized microglia highly expressed CD64, and moderately expressed CD32 (Fig. 6). We do not know whether the low expression of CD16 detected is due to either the quality of the antibody or to the fact that these immortalized microglial cells have low level of this Fc receptor, since we did not test a positive control side by side.

### Inflammatory responses of microglial cells

A recently published study on primary cells originating from adult human brain (Darmanis et al. 2015) used single-cell RNA-Seq to classify individual cells into the major neuronal, glial, and vascular cell types found in the brain. We took advantage of this resource to further evaluate whether our immortalized human microglial cells (clone C20) retained a microglial phenotype. As shown in Fig. 8, the gene expression profile of both untreated and TNF-α-treated C20 cells showed strong similarities with and clustered more closely with primary microglia than to the other brain cell types examined, further proving evidence of their microglial origin.

The RNA-Seq experiment also revealed that an inflammatory stimulus (TNF-α), induced major pro-inflammatory pathways (Tables 1 and 2) characteristic of macrophages and microglia, and reported to be implicated in a number of neurodegenerative disorders (Dominguez et al. 2008; Fonken et al. 2015; Kaltschmidt et al. 2005; Khorooshi et al. 2015; Kigerl et al. 2014; Kothur et al. 2016; Sheng et al. 2014; Tsuda et al. 2009).

Microglial cells are a major source of cytokines and chemokines necessary for the regulation of immune responses in CNS. Activation of inflammatory pathways observed through increased of transcript levels of pro-inflammatory molecules (Fig. 8b; Tables 1 and 2) in TNF-α-stimulated C20 cells was corroborated by the measurement of secreted cytokines and chemokines released, another criterion we have used to characterize our immortalized microglial cell lines.

Upon administration of pro-inflammatory stimuli, microglia may release different combinations of cytokines that include interferons, interleukins, TGF-β, GM-CSF, PDGF, EGF, FGF, IGF, NGF, neurotrophins, and BDNF (Hanisch 2002). Likewise, under inflammatory conditions, microglia may also secrete different types of chemokines, including MIP-1α, MIP-1β, and MCP-1 (Lee et al. 2002). As shown in Fig. 9, stimulated C20 cells released microglia-specific key mediators of inflammation at different levels. IL-8, which has been reported to be increased in plasma, serum, and cerebrospinal fluid of HIV patients (Carrol et al. 2007; Lane et al. 2001), GROα, which is involved in neutrophil infiltration during brain injury and inflammation (Johnson et al. 2011), GM-CSF, which upregulates TLR4 and CD14 expression in microglia promoting LPS-mediated inflammation in the CNS (Parajuli et al. 2012), IL-6, which has been implicated in the development of various neurodegenerative processes (Spooren et al. 2011), and MCP-1, a major player in the migration and proliferation of microglia (Hinojosa et al. 2011), were among the highest secreted molecules (Fig. 9).

Similarly, in the mouse cells, we detected activation of the pro-inflammatory genes TNF-α, IL-1β, and IL-6 upon stimulation of our microglial cells with LPS (Fig. 10d), a hallmark of microglial cells.

### Misidentification of CHME-5 cells

CHME-5 cells (Janabi et al. 1995) have been erroneously classified as SV40-immortalized microglial cells of human origin, even in very recent studies (Lisi et al. 2015; Wires et al. 2012). However, genetic studies revealed that these cells are of rat origin and do not express the SV40 T antigen (Fig. 3b), rendering them inappropriate as a model of human microglia. The fact that they do not carry the SV40 T antigen suggests that the cells in circulation in numerous laboratories are in fact a rat glioblastoma which may have been an early contaminant of the original cells. We have been unable to identify any sources of CHME-5 cells that are of human origin.

Previous studies of HIV in microglial cells (Chugh et al. 2007; Jadhav et al. 2014; Janabi et al. 1998; Martin et al. 2003; Wires et al. 2012), including some of our own, have been compromised because the cells were either derived from CHME-5 or used incompletely characterized cell lines.

The risk of cross contamination between cells from different species increases when multiple cell lines are passage in the same laboratory. In order to avoid species cross contamination, we now routinely verify the cell cultures by amplification of the CYCT1 gene with specific primers designed to target mouse, rat, macaque or human CYCT1.

### Development of microglial cell models for HIV latency

Using these immortalized cells, we have been able establish powerful cellular models to study HIV latency and regulation (Fig. 11) using methods similar to those described previously for CHME-5 cells (Wires et al. 2012). The latently infected cells show minimal expression of HIV, but can be readily activated by TNF-α and other stimuli. In a companion paper, we have used these latently infected cells to show how TLR signaling mediates HIV reactivation in microglia and how TLR3, unlike other TLRs, plays a preponderant role in mediating pathogen-induced HIV reactivation in microglia (Alvarez-Carbonell et al. 2016).

In conclusion, we have shown here that freshly isolated microglial cells from brain tissue can be productively infected with HIV, can be immortalized, and after immortalization, retain major phenotypic and functional features of primary microglia. The repeated observations that resident brain macrophages are highly susceptible to HIV infection, strongly suggests that HIV-related neurologic disorders may be the result of not only HIV-infected cells that infiltrated into CNS from the periphery but also of HIV-infected microglia. The reliable cellular models for microglial cells we report here provide a strong experimental system to study key molecular events associated with various microglia-related neurodegenerative disorders, including HAND.

## Electronic supplementary material

Supplementary Figure 1 Anti-CD4 and -CCR5 antibodies (X-axis) were used to quantify surface levels of expression by flow cytometry (Y-axis) on 4 different cell culture passages, as indicated, of C20 cells. (PDF 290 kb)

**Dataset S1:** Excel file with sets of genes for the hallmark pathways related to the pro-inflammatory responses which are increased in hμglia C20 cells treated with TNF-α (*p* ≤ 0.001), as determined by the Gene set Enrichment Analysis (GSEA). (XLSX 123 kb)

**Dataset S2:** Generic and genes graphs generated by the Kyoto Encyclopedia of Genes and Genomes (KEGG) depicting the proteins (generic graphs) or genes (gene graphs) found up-regulated in hμglia C20 cells treated with TNF-α, grouped in six different pathways, as listed in Table 2. (GIF 26 kb)

High resolution image (TIFF 94 kb)

ESM 3 (GIF 33 kb)

High resolution image (TIFF 163 kb)

ESM 4 (GIF 62 kb)

High resolution image (TIFF 146 kb)

ESM 5 (GIF 78 kb)

High resolution image (TIFF 341 kb)

ESM 6 (GIF 42 kb)

High resolution image (TIFF 225 kb)

ESM 7 (GIF 54 kb)

High resolution image (TIFF 151 kb)

ESM 8 (GIF 32 kb)

High resolution image (TIFF 121 kb)

ESM 9 (GIF 40 kb)

High resolution image (TIFF 182 kb)

ESM 10 (GIF 31 kb)

High resolution image (TIFF 93 kb)

ESM 11 (GIF 40 kb)

High resolution image (TIFF 166 kb)
