# Supplementary figures and images for: Immortalization of primary microglia: a new platform to study HIV regulation in the central nervous system
Source: J Neurovirol. 2016 Nov 21;23(1):47–66. doi: 10.1007/s13365-016-0499-3 (PMC5329090; doi:10.1007/s13365-016-0499-3)

# CD4 and CCR5 Across Cell Culture Passages

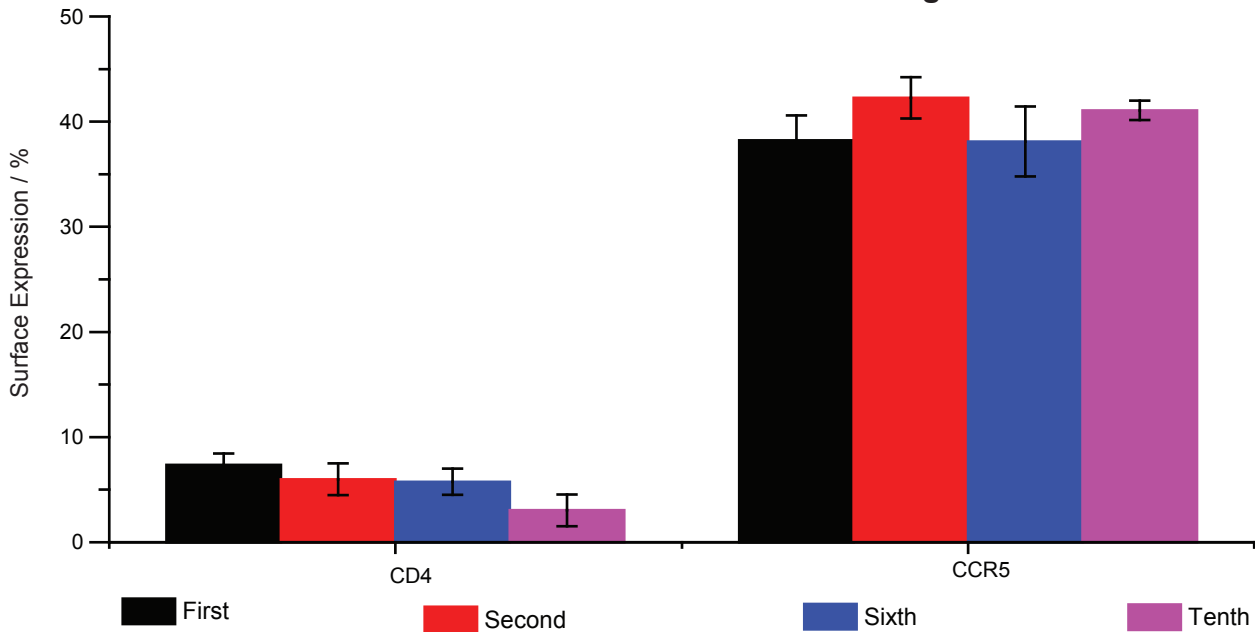

Supplement: Supplementary file 1 — Anti-CD4 and -CCR5 antibodies (X-axis) were used to quantify surface levels of expression by flow cytometry (Y-axis) on 4 different cell culture passages, as indicated, of C20 cells. (PDF 290 kb) [file 13365_2016_499_Fig12_ESM.pdf]

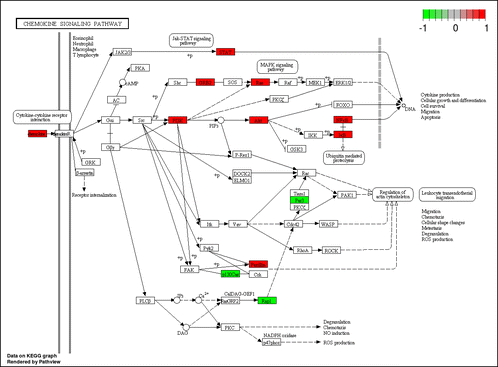

Supplement: Supplementary file 3 — Dataset S2: Generic and genes graphs generated by the Kyoto Encyclopedia of Genes and Genomes (KEGG) depicting the proteins (generic graphs) or genes (gene graphs) found up-regulated in hμglia C20 cells treated with TNF-α, grouped in six different pathways, as listed in Table 2. (GIF 26 kb) [file 13365_2016_499_Fig13_ESM.gif]

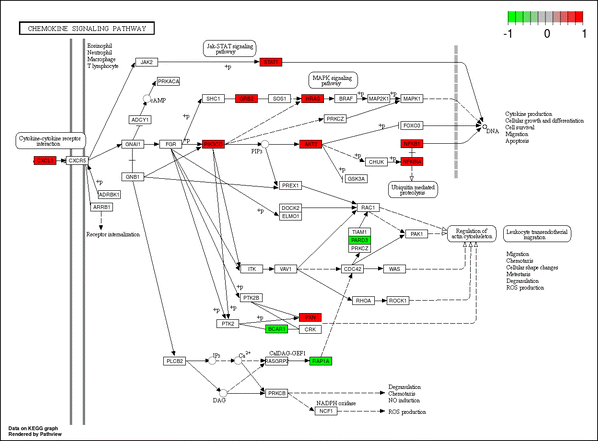

Supplement: Supplementary file 5 — (GIF 33 kb) [file 13365_2016_499_Fig14_ESM.gif]

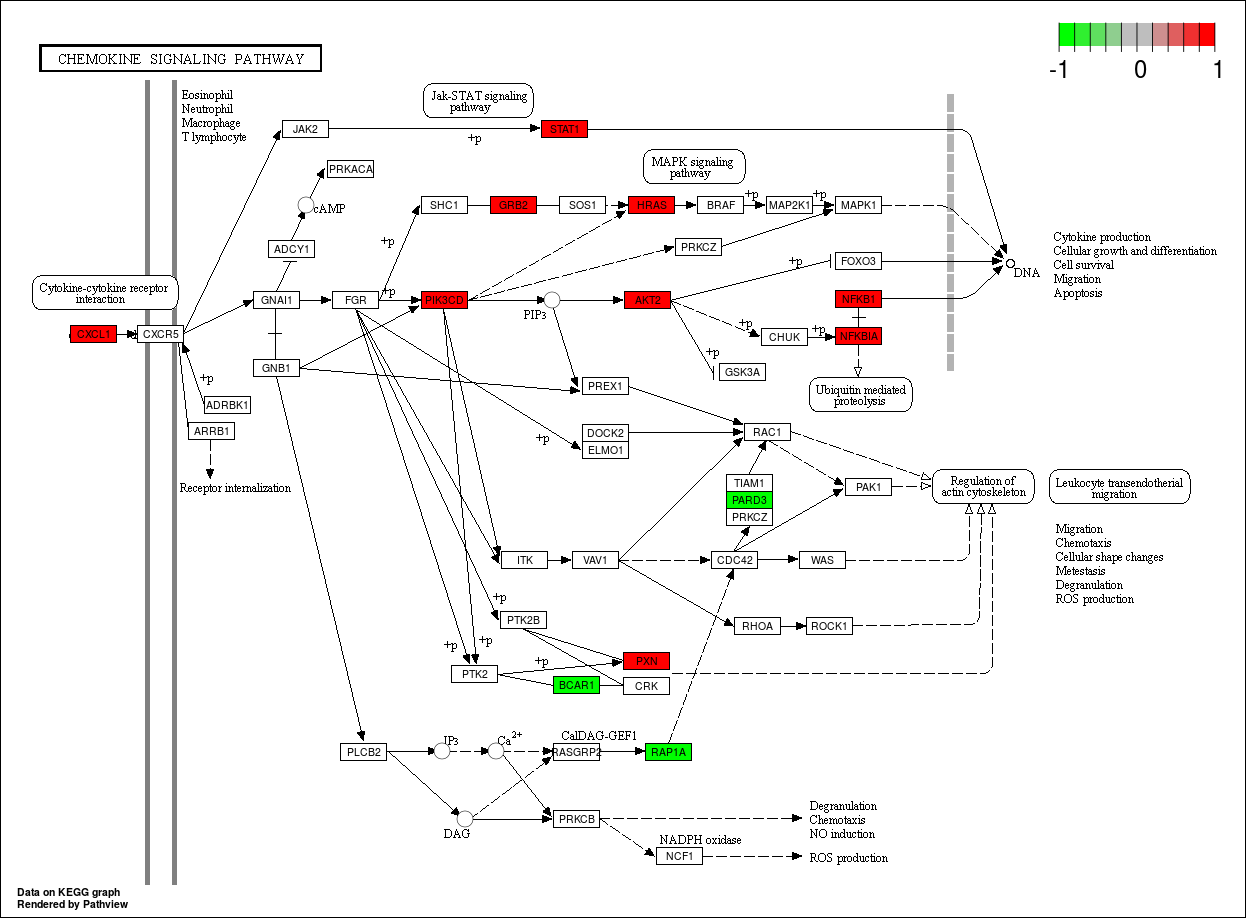

Supplement: Supplementary file 6 — High resolution image (TIFF 163 kb) [file 13365_2016_499_MOESM3_ESM.tif]

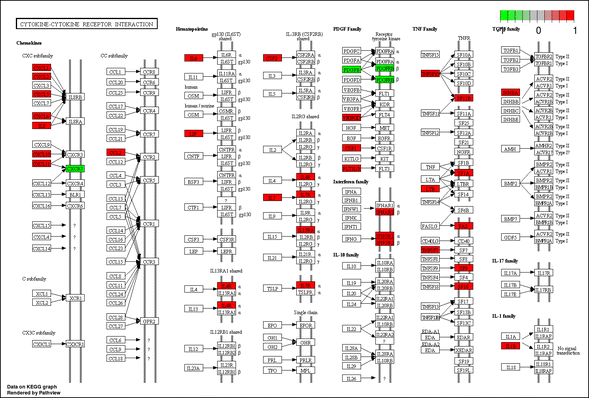

Supplement: Supplementary file 7 — (GIF 62 kb) [file 13365_2016_499_Fig15_ESM.gif]

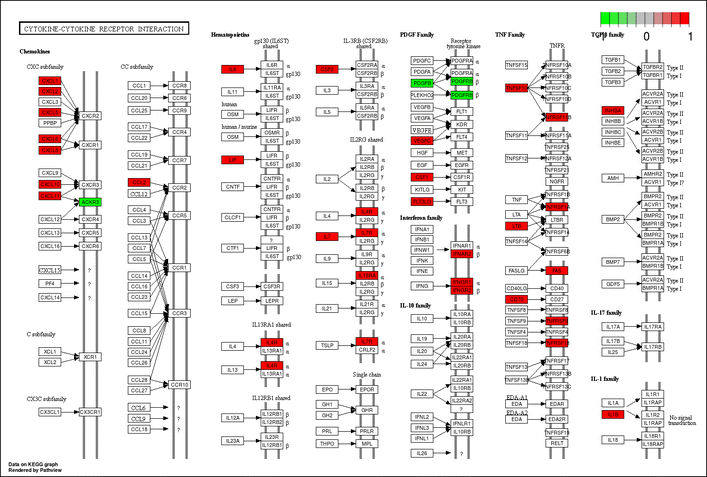

Supplement: Supplementary file 9 — (GIF 78 kb) [file 13365_2016_499_Fig16_ESM.gif]

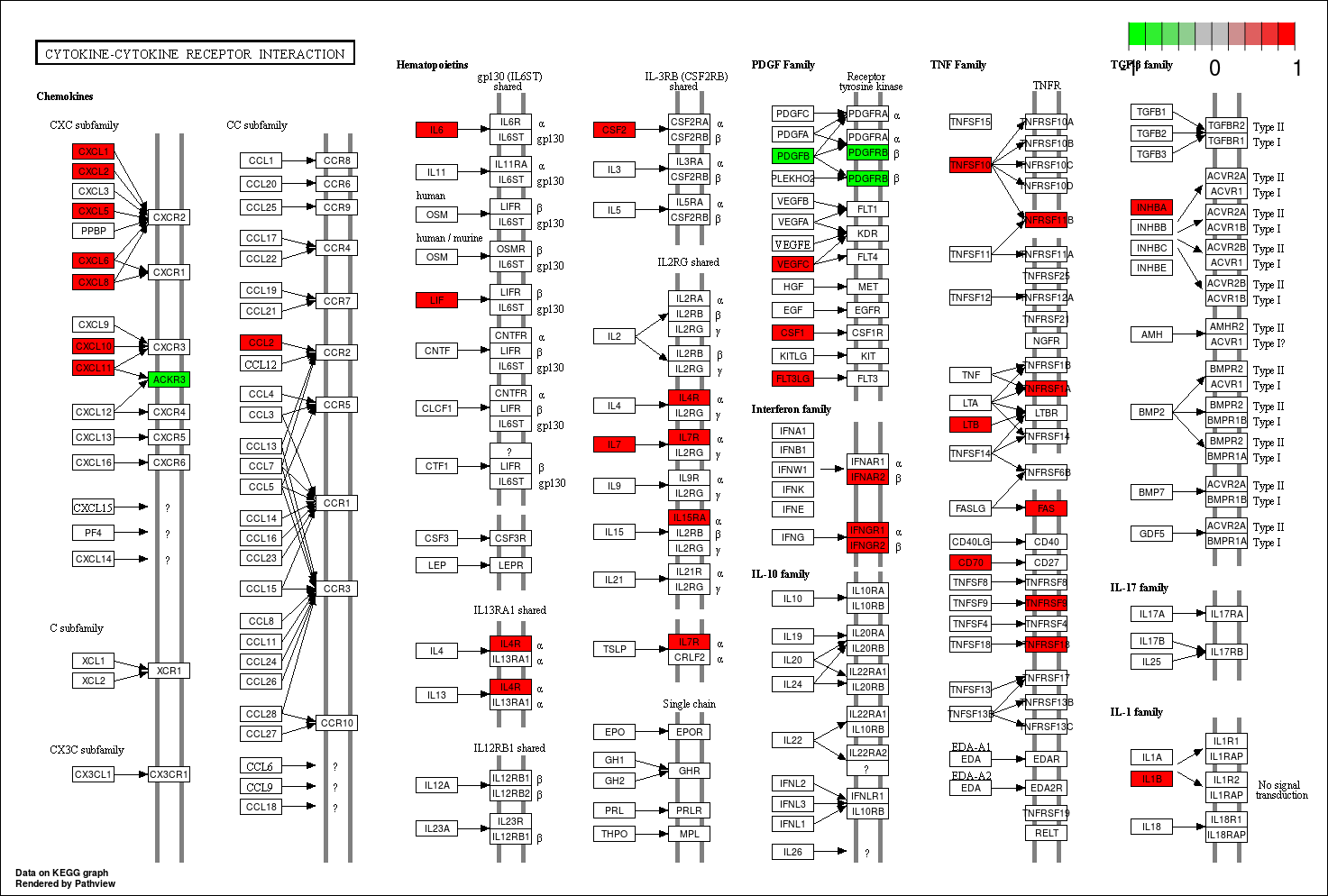

Supplement: Supplementary file 10 — High resolution image (TIFF 341 kb) [file 13365_2016_499_MOESM5_ESM.tif]

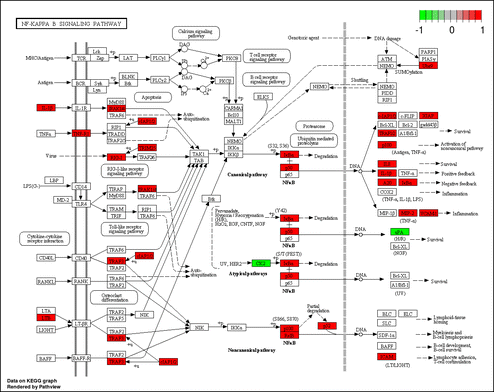

Supplement: Supplementary file 11 — (GIF 42 kb) [file 13365_2016_499_Fig17_ESM.gif]

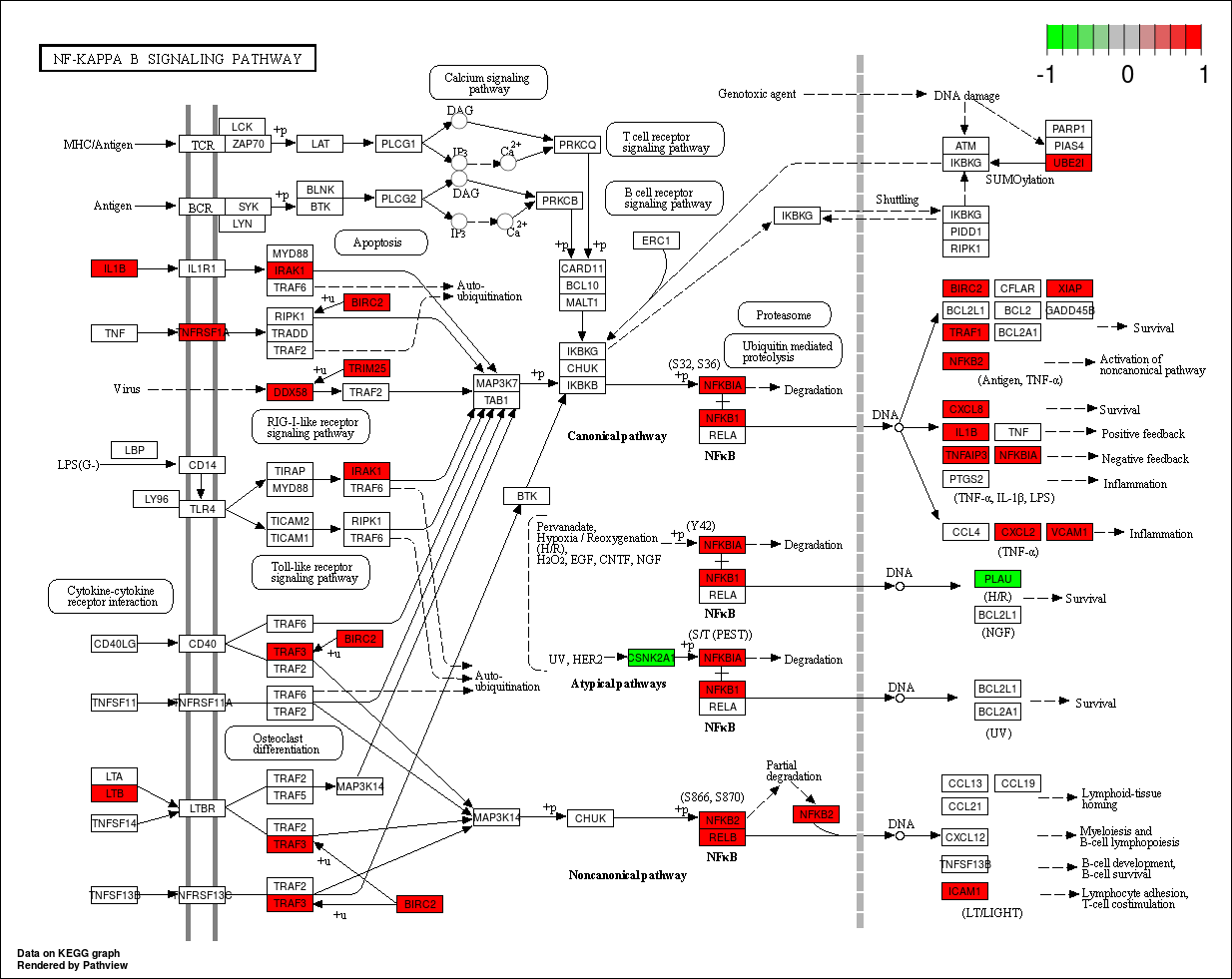

Supplement: Supplementary file 12 — High resolution image (TIFF 225 kb) [file 13365_2016_499_MOESM6_ESM.tif]

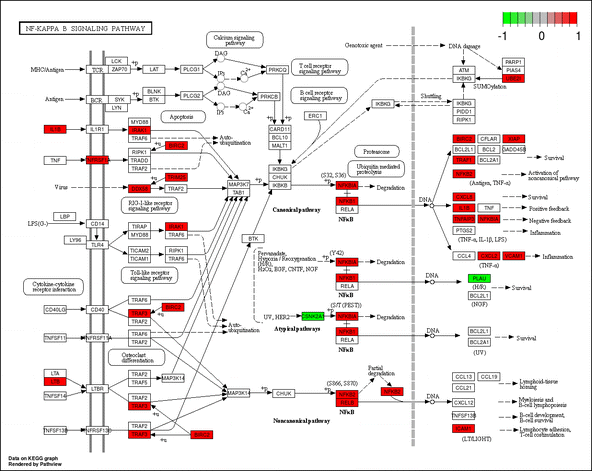

Supplement: Supplementary file 13 — (GIF 54 kb) [file 13365_2016_499_Fig18_ESM.gif]

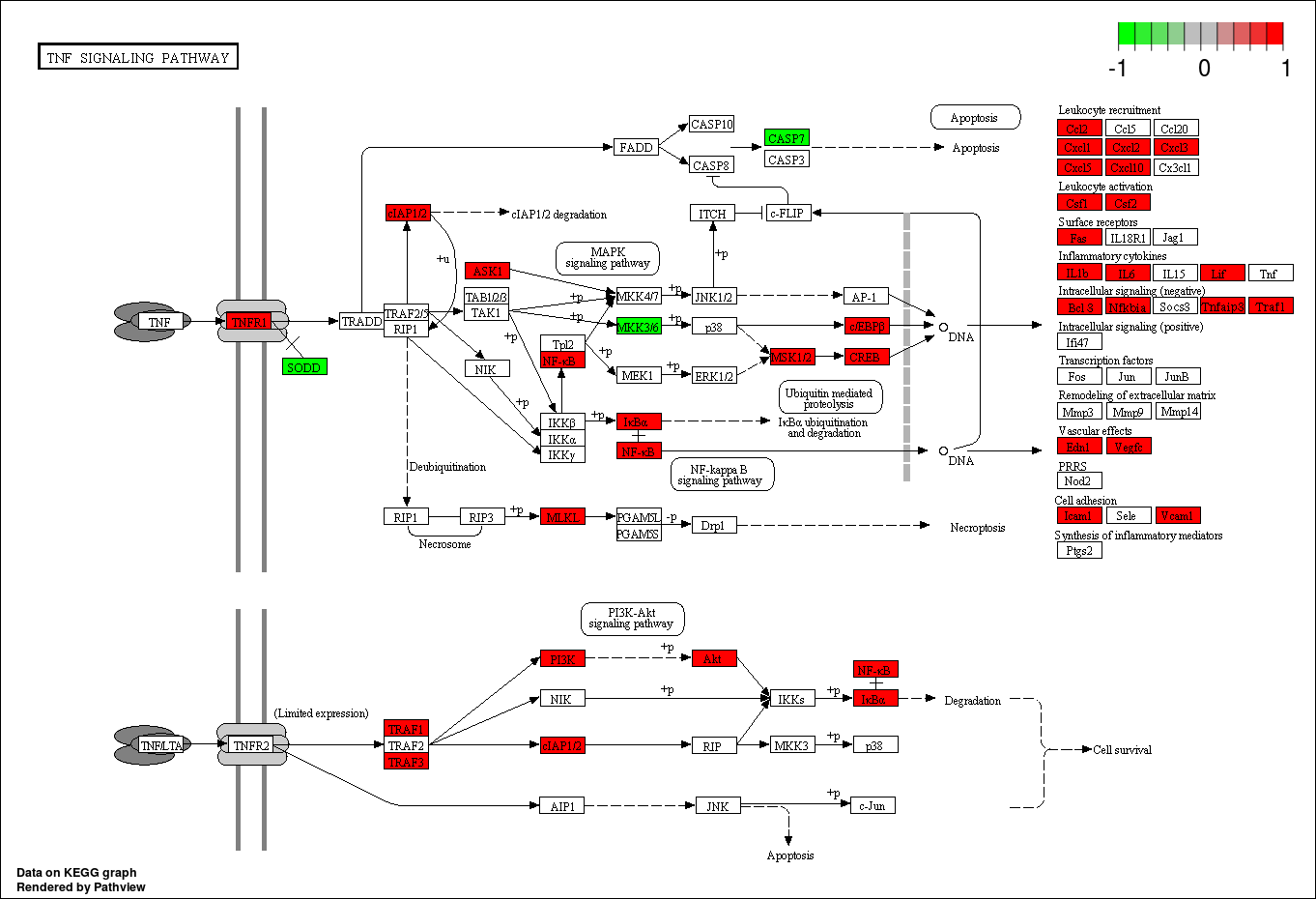

Supplement: Supplementary file 14 — High resolution image (TIFF 151 kb) [file 13365_2016_499_MOESM7_ESM.tif]

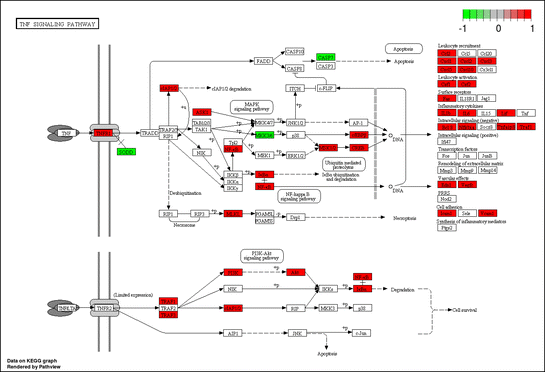

Supplement: Supplementary file 15 — (GIF 32 kb) [file 13365_2016_499_Fig19_ESM.gif]

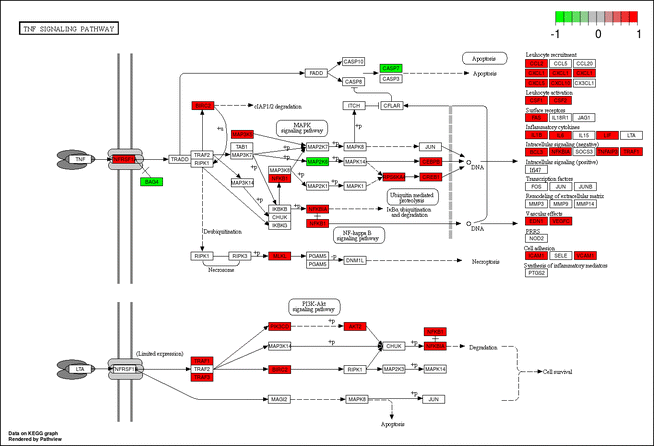

Supplement: Supplementary file 17 — (GIF 40 kb) [file 13365_2016_499_Fig20_ESM.gif]

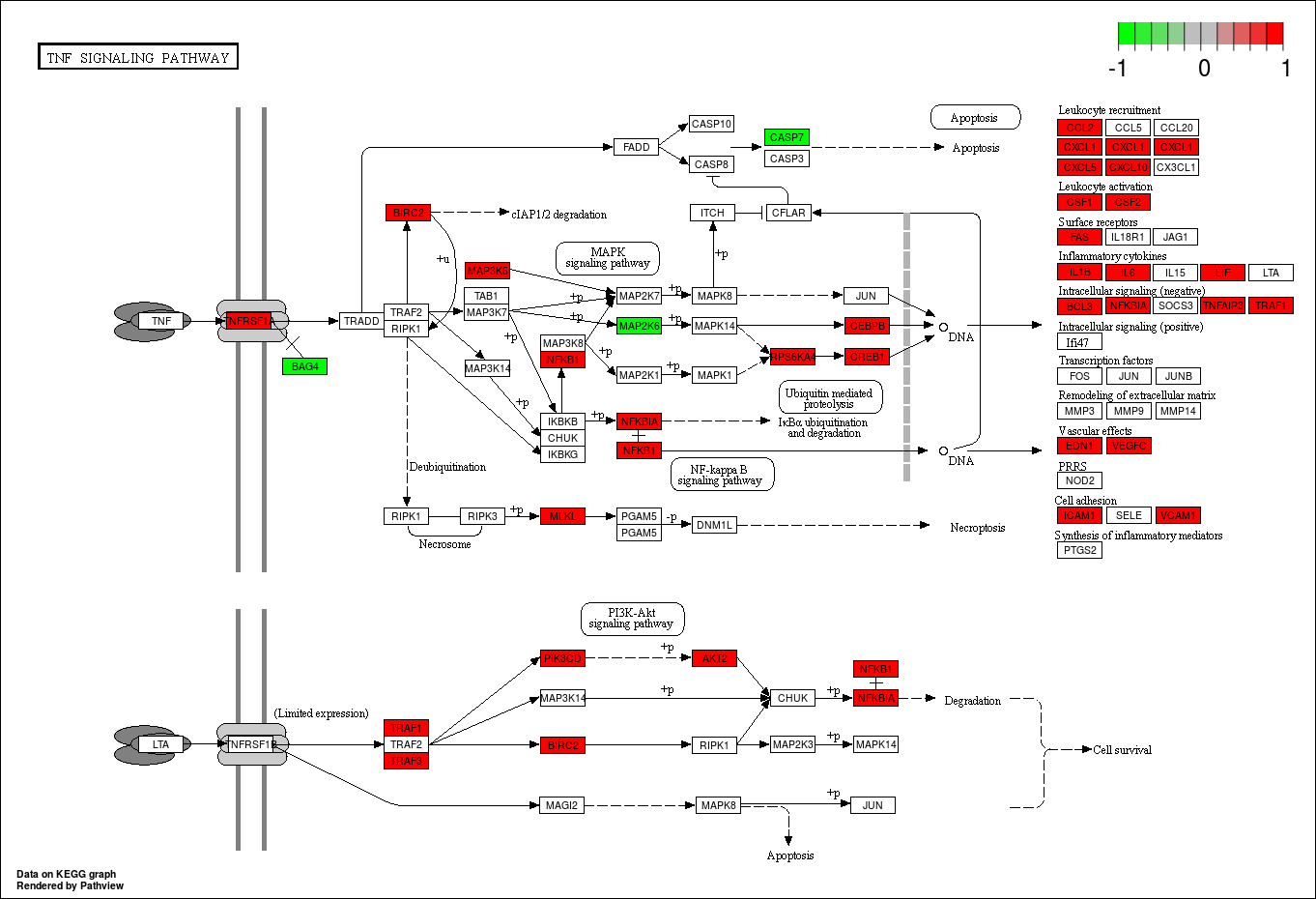

Supplement: Supplementary file 18 — High resolution image (TIFF 182 kb) [file 13365_2016_499_MOESM9_ESM.tif]

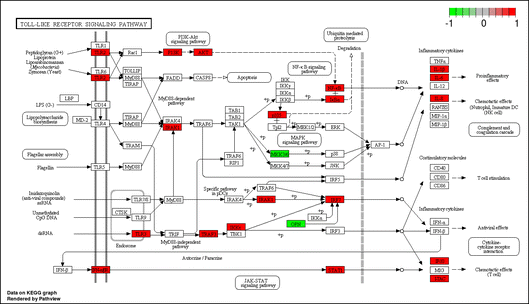

Supplement: Supplementary file 19 — (GIF 31 kb) [file 13365_2016_499_Fig21_ESM.gif]

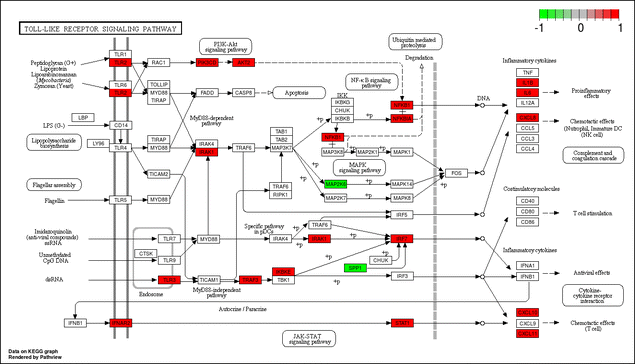

Supplement: Supplementary file 21 — (GIF 40 kb) [file 13365_2016_499_Fig22_ESM.gif]

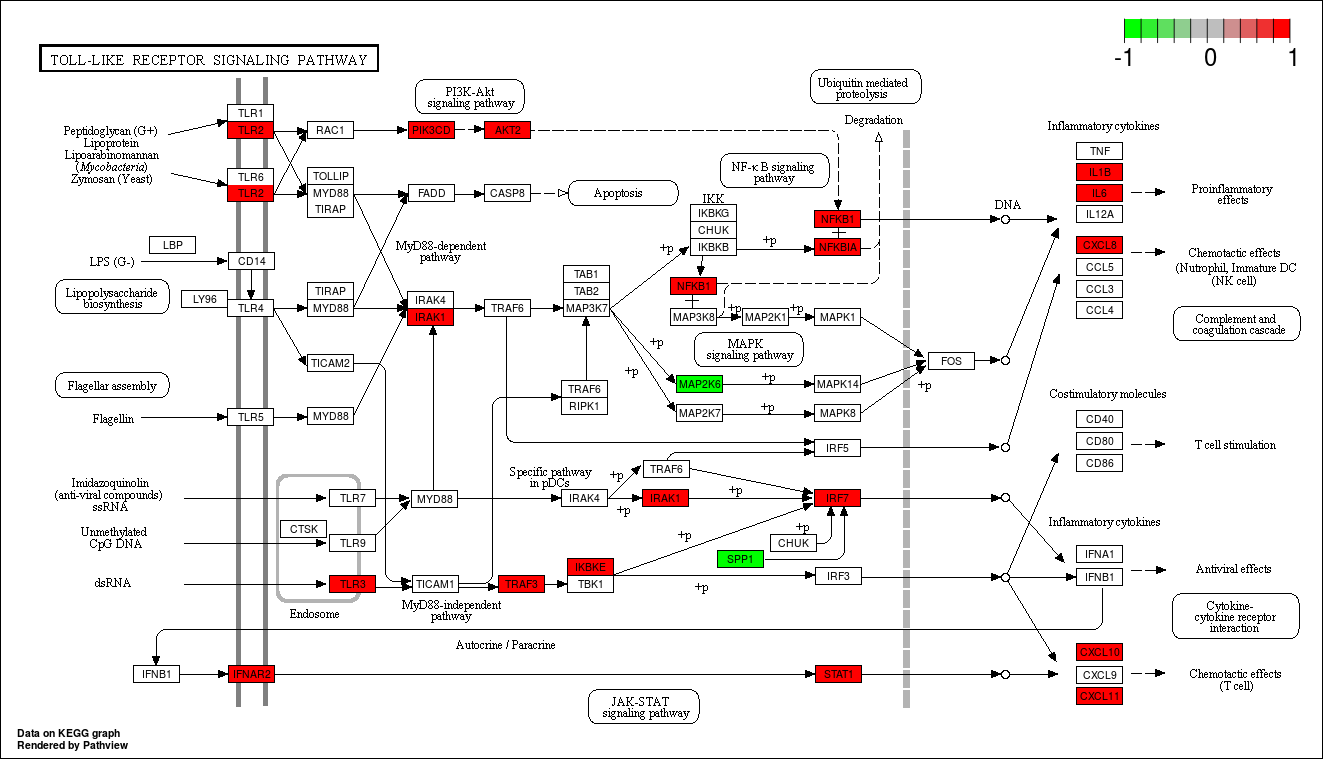

Supplement: Supplementary file 22 — High resolution image (TIFF 166 kb) [file 13365_2016_499_MOESM11_ESM.tif]
